# People do change their beliefs about conspiracy theories—but not often

**DOI:** 10.1038/s41598-024-51653-z

**Published:** 2024-02-15

**Authors:** Matt N. Williams, Mathew Ling, John R. Kerr, Stephen R. Hill, Mathew D. Marques, Hollie Mawson, Edward J. R. Clarke

**Affiliations:** 1 Massey University, North Shore, Private Bag 102904, Auckland, 0745 New Zealand; 2 Deakin University, Geelong, Australia; 3 Neami National, Preston, Australia; 4 University of Cambridge, Cambridge, UK; 5 University of Otago, Wellington, New Zealand; 6 Massey University, Palmerston North, New Zealand; 7 La Trobe University, Melbourne, Australia; 8 Philipps-Universität Marburg, Marburg, Germany

**Keywords:** Human behaviour, Psychology and behaviour

## Abstract

Recent research has produced a significant body of knowledge about the antecedents and consequences of individual differences in belief in conspiracy theories. What is less clear, however, is the extent to which individuals’ beliefs in conspiracy theories vary over time (i.e., *within-person* variation). In this descriptive and exploratory study, we therefore aimed to describe within-person variability in belief in conspiracy theories. We collected data from 498 Australians and New Zealanders using an online longitudinal survey, with data collected at monthly intervals over 6 months (March to September 2021). Our measure of conspiracy theories included items describing ten conspiracy theories with responses on a 5-point Likert scale. While there was substantial between-person variance, there was much less within-person variance (intraclass *r* = 0.91). This suggests that beliefs in conspiracy theories were highly stable in our sample. This stability implies that longitudinal studies testing hypotheses about the causes and consequences of belief in conspiracy theories may require large samples of participants and time points to achieve adequate power. It also implies that explanations of belief in conspiracy theories need to accommodate the observation that beliefs in such theories vary much more between people than within people.

## Introduction

Recently there has been an explosion of interest in conspiracy theories within psychology. Researchers have sought to better understand the prevalence, causes and consequences of belief in conspiracy theories^1,2^. Much of this research has focused on *individual differences*: To what extent do beliefs in conspiracy theories vary across individuals, and why? Much less research, however, has focused on describing the degree to which beliefs in conspiracies change over time *within* individuals. Description has important intrinsic value in the scientific process: By detecting and describing phenomena we can then proceed to explaining, predicting, and changing those phenomena^3^.

### Existing longitudinal research

A small number of longitudinal studies on beliefs in conspiracy theories have already been published. Most of these studies have had aims other than to describe variability in beliefs over time per se. Nevertheless, it is possible to derive some qualified information about the within-person (intra-individual) variability of belief in conspiracies over time from these studies.

Several studies have reported correlations between individuals’ beliefs in conspiracy scores at two time points, which speak to the stability of individual participants’ beliefs. Romer and Jamieson^4^ reported a survey across two time points spanning 4 months which included three specific COVID-19 conspiracy theory items. They found a slight increase in mean scores on these items over time and a large correlation between pre and post scores (*r* = 0.75). Jolley et al.^5^ examined the link between conspiracy theory beliefs and political decision making in relation to support for Brexit at two time points a week apart, before and after the referendum. They found that beliefs in conspiracy theories about Brexit at times 1 and 2 were strongly correlated (*r* = 0.78), although the mean level of belief declined slightly after the referendum. These strong correlations suggest a relatively low degree of within-person variability in beliefs.

Other studies have incorporated more than two time points. An investigation of belief in conspiracy theories about the origins of COVID-19 across five time points in a sample of 403 US residents^6^ between March and April 2020 found strong relationships between beliefs in conspiracy theories across different time points (*r*s between 0.67 and 0.84). A study with slightly fewer waves (four), examined the relationship between COVID-19 conspiracy theory beliefs and self-reported health protective behaviour in a representative sample of Polish citizens between May and December 2020^7^. This study again found strong correlations over time, albeit these correlations were slightly smaller when the time points were further apart.

Another study with four waves^8^ investigated “conspiracy thinking” during the US presidential campaign between July and November 2016. The items used in this study (e.g., “The people who really ‘run’ the country are not known to the voters”; p. 1013) would typically be described as measures of *conspiracy mentality*—i.e., “generalized worldviews suspecting conspiracy at play”^9^. The authors found a moderately strong correlation between conspiracy thinking at the endpoints of July and November (*r* = 0.56).

In one of the most relevant studies for our purposes, Liekefett et al.^10^ used random intercepts cross-lagged panel models (RI-CLPMs) to examine the associations between general and COVID-19 specific conspiracy theory beliefs alongside other variables (anxiety, uncertainty aversion, and existential threat) over two studies, spanning 6 weeks and 12 months respectively (each with four time points). A valuable feature of their article was their use of intraclass correlation coefficients (ICCs) to explicitly estimate the proportion of variability in conspiracy beliefs that could be attributed to stable individual differences. In study 1, the intraclass coefficient (ICC) of 0.80 for general conspiracy beliefs indicated that just 20% of variation in conspiracy beliefs over the course of the study could be attributed to within-person variability as opposed to stable between-person differences. In study 2, this intraclass correlation was only slightly lower at 0.75 despite the longer time period (12 months). In a similar study, Coelho et al.^11^ administered a measure of vaccination conspiracy beliefs with a sample of 500 Australian adults five times over 4 months in 2021, finding an ICC of 0.83.

In summary, there is some evidence suggesting that beliefs in conspiracy theories are fairly—but not extremely—stable over time. Nevertheless, none of the studies discussed above have focused specifically on a detailed exploration and quantification of the magnitude of change over time within individuals, instead having largely focused their statistical reporting on relationships between variables. The existing longitudinal studies have also collected data over relatively few time points (often just two; maximum of five) and generally focused only on specific subtypes of conspiracy theories (e.g., COVID-19 conspiracy theories). This means that they are somewhat limited sources for characterising change over time.

### Aims of the current study

In this study we therefore aim to investigate the degree of within-person variability of belief in conspiracy theories in a sample of participants surveyed at seven data collection points, each spaced 1 month apart from March to September 2021. In doing so we will take a descriptive and exploratory approach^12,13^ rather than testing any specific hypothesis or theory.

We collected these data from residents of Australia and New Zealand against the backdrop of the second year of the COVID-19 pandemic. This was a period in which both countries faced the emergence of the Delta variant of the SARS-COV-2 virus, with resulting lockdowns, while simultaneously rolling out vaccines to the general population (for a timeline see^14^).

## Method

### Design

This study used a longitudinal survey design, wherein the same cohort of participants were invited to complete surveys on seven occasions over 6 months. The open and close dates for each survey are listed in Table 1.

**Table 1:** Dates of survey waves.

Wave	Survey open and close dates	N after exclusions	Percent of original N
1	19–19 March 2021^a^	498	100
2	19–26 April 2021	432	87
3	19–26 May 2021	379	76
4	19–26 June 2021	377	76
5	19–26 July 2021	370	74
6	19–26 August 2021	360	72
7	19–26 September 2021	331	66

^a^The wave 1 survey met its quota much more quickly than the remaining waves due to being open to a wide sampling frame. Subsequent surveys were open only to those who had completed a survey at time 1, necessitating longer open periods.

### Sample size determination

This study does not involve tests of hypotheses or an emphasis on significance testing, meaning that statistical power analysis was not a suitable tool for sample size determination. Instead, the target sample size (approximately *N* = 500) was determined by practical and financial constraints. See the Supplementary Information for a precision analysis.

### Participants and procedure

The participants were adults (18+) currently living in New Zealand or Australia. Eligibility was limited to these two countries for several reasons. First, it permitted recruitment from outside the traditionally oversampled regions of North America and Europe. Second, most of the authors are located in Australia and New Zealand, meaning that it was feasible for us to develop conspiracy theory items that would be understandable to participants and relevant to the local cultural milieu. Third, the combination of these two countries provided a sufficiently large sampling frame when using the selected recruitment platform.

Participants were recruited from Prolific, a popular crowdsourcing platform for behavioural research^15^ that has been found to facilitate high retention rates in longitudinal studies^16^. Prolific allows for participants to be “prescreened” on a range of criteria. For the initial recruitment (the first survey), the only prescreening criterion was that participants’ current country of residence had to be Australia or New Zealand. We specified a target sample size of 500 at time 1; ultimately 503 responses were received. The number of responses received when using Prolific often slightly exceeds the target sample size, due to some participants filling out a survey but not entering a completion code.

Within the first survey, we included an open-ended attention check: “What is your favourite sport, and why is it your favourite?” Just three participants were excluded due to providing incomplete responses (e.g., naming a sport without a reason). Two further participants were excluded for not completing all the conspiracy theory items.

The remaining sample of *N* = 498 from time 1 were then invited to complete follow-up surveys at monthly intervals. At each wave, the survey was made available for a period of 1 week (commencing at monthly intervals from the start date of the time 1 survey). Within the available period for each survey, the first author sent a reminder message via the Prolific system to each participant who had not yet responded. If a participant missed a wave they were still invited to subsequent waves.

Participants were paid GBP0.50 for the survey at time 1 (which was slightly longer due to the qualitative attention check and some demographic questions), then GBP0.40 for times 2 through 6. They were paid a slightly larger payment of GBP0.60 as a reward for the final time point.

52.4% of participants described their gender as male, 46.8% as female, and 0.8% as non-binary or gender diverse. The mean age was 32.8 years (*SD* = 10.7). Participants were generally highly educated; 66.3% had at least an undergraduate degree. Most participants were either politically liberal (45.6%) or moderate (42.0%). Just 8.8% described themselves as conservative. More detailed demographic information can be found in Supplementary Table 1.

### Ethics

This study was deemed to be low risk according to the criteria of Massey University. A low-risk notification (similar to an “exempt review” application in North America) was therefore lodged and accepted (#4000024064). The study was conducted in accordance with the Massey University code of ethical conduct for research, teaching and evaluations involving human participants^17^. All participants provided informed consent (by reading an information sheet and then answering a consent item).

### Exclusion criteria

The criteria used to determine the sample to be invited back after time 1 are described above. All but one of the subsequent surveys (time 2) included a simple attention check, wherein participants were explicitly directed to select a specific option. Individual surveys within which a participant failed an attention check were excluded from analyses, but the participant was still retained in the sample for subsequent invites. Duplicate responses from the same participant within a single time point (as identified using Prolific ID number) were also deleted, as were any responses from participants who revoked their consent by “returning” their submissions in Prolific.

### Measure

Beyond a selection of demographic items, the only measure of interest in the current study was our set of conspiracy theory items. We chose not to use a pre-existing measure of belief in conspiracy theories such as the Belief in Conspiracy Theories Inventory^18^ to ensure that the conspiracy theories presented in our items were of contemporary relevance. This increased the plausibility that we might see fluctuations in belief over time. We constructed and adapted items to describe notable conspiracy theories pertaining to claimed events which are either ongoing or occurred in the last 20 years (at the time of data collection). For further information about the process we used to construct items, see the Supplementary Information.

We included two conspiracy theories that are warranted by empirical evidence (the Watergate and MK-Ultra theories; see Bernstein and Woodward, 1974, and Committee on Human Resources, 1977). These items were included partly to maintain participants’ attention via some diversity of content. For brevity, we have not reported analyses using these items in the sections below, but readers can find these analyses in the Supplementary Information.

We used a balanced response format for each question, with five options ranging from strongly disagree (1) to strongly agree (5). For a discussion of the benefits of this approach in the context of measuring belief in conspiracy theories, see^19^. We created a conspiracy belief score for each participant at each time point by calculating the mean of their responses to all items at that time point (possible range 1 to 5). Cronbach’s alpha at time 1 was 0.86.

### Attrition and missing data

The number of participants completing surveys at each time point gradually declined over the course of the study (see Table 1 above). This attrition was not severe, with 66% of eligible participants from time 1 still participating at the final data collection point (time 7). 472 participants (95%) responded to at least two waves. There was just a single missing data point at the item level *within* the surveys that were attempted by participants. Missing data points were excluded from the reported analyses. Participants with higher levels of belief in conspiracy theories were somewhat more likely to return for more waves; see the Supplementary Information for an examination of predictors of retention.

### Statistical assumptions

Examinations of the validity of the assumptions underlying our analyses can be found in our Supplementary Information.

## Results

Our focus in this study is on within-person variance in beliefs about conspiracy theories. However, the quantity of this within-person variance is best expressed relative to the quantity of variance attributable to the conspiracy theories (inter-theory variation) and to stable individual differences (“inter-individual” or between-person variation). We therefore begin with a brief examination of these sources of variation.

### Inter-theory variation

To investigate the quantity of variation in beliefs across theories we calculated the mean response to each theory (across all participants and waves; see Table 2). Participants tended to disagree with the presented theories; the mean response for each of the theories was below the scale midpoint of 3. The most popular conspiracy theory was that pharmaceutical companies have suppressed a cure for cancer (*M* = 2.23). Overall, the standard deviation of mean responses across theories was 0.31.

**Table 2:** Conspiracy theory items.

Abbreviation	Item	Source	T1% agree^a^	T7% agree	M (SD)^b^
COVID = weapon	COVID-19 is a biological weapon intentionally created and released by China	Adapted from Miller^20^	11.8	14.2	1.96 (1.17)
COVID vax chip	COVID-19 “vaccines” contain microchips to monitor and control people	Constructed for this study	1.6	0.9	1.24 (0.61)
Vaccines	Vaccines are harmful, and this fact is covered up by governments and pharmaceutical companies	Adapted from Jolley and Douglas^21^	5.8	8.8	1.67 (0.97)
Trump	Democrats stole the 2020 US Presidential election from Donald Trump by creating fraudulent ballots	Paraphrased from the Wikipedia page^22^	9.2	10.9	1.66 (1.14)
NWO	A powerful and secretive group, known as the New World Order, are planning to rule the world	Adapted from Swami et al.^18^	13.7	13.9	1.93 (1.14)
5G	Telecommunication companies are covering up the health risks of the new 5G cellular network	Adapted from Marques et al.^23^	11.6	10.3	1.77 (1.10)
Chemtrails	The trails left behind airplanes are toxic chemicals released as part of a secret government programme	Adapted from Oliver and Wood^24^	3.2	3.0	1.42 (0.78)
Fluoride	Fluoride is added to the water supply by governments to make people less intelligent and easier to control	Adapted from Marques et al.^23^	3.4	3.6	1.42 (0.82)
911	The collapse of the World Trade Centre on Sept 11, 2001 was caused by controlled demolitions arranged by US government insiders	Constructed for this study	14.1	14.8	2.05 (1.25)
Cancer cure	Pharmaceutical companies ("Big Pharma") have suppressed a cure for cancer to protect their profits	Constructed for this study	17.9	20.2	2.23 (1.27)

^a^(Agree + strongly disagree)/all.

^b^Means and standard deviations calculated across all participants and waves; possible range 1 to 5.

### Between-person variation

To describe between-person variation in responses, we calculated each participant’s mean response across all theories and waves. Participant means ranged from 1.00 to 4.37, with a grand mean of 1.71. Most importantly, the standard deviation of the mean response across participants was 0.73, suggesting a relatively substantial quantity of between-person variation.

### Change in mean agreement over time

**Figure 1:**
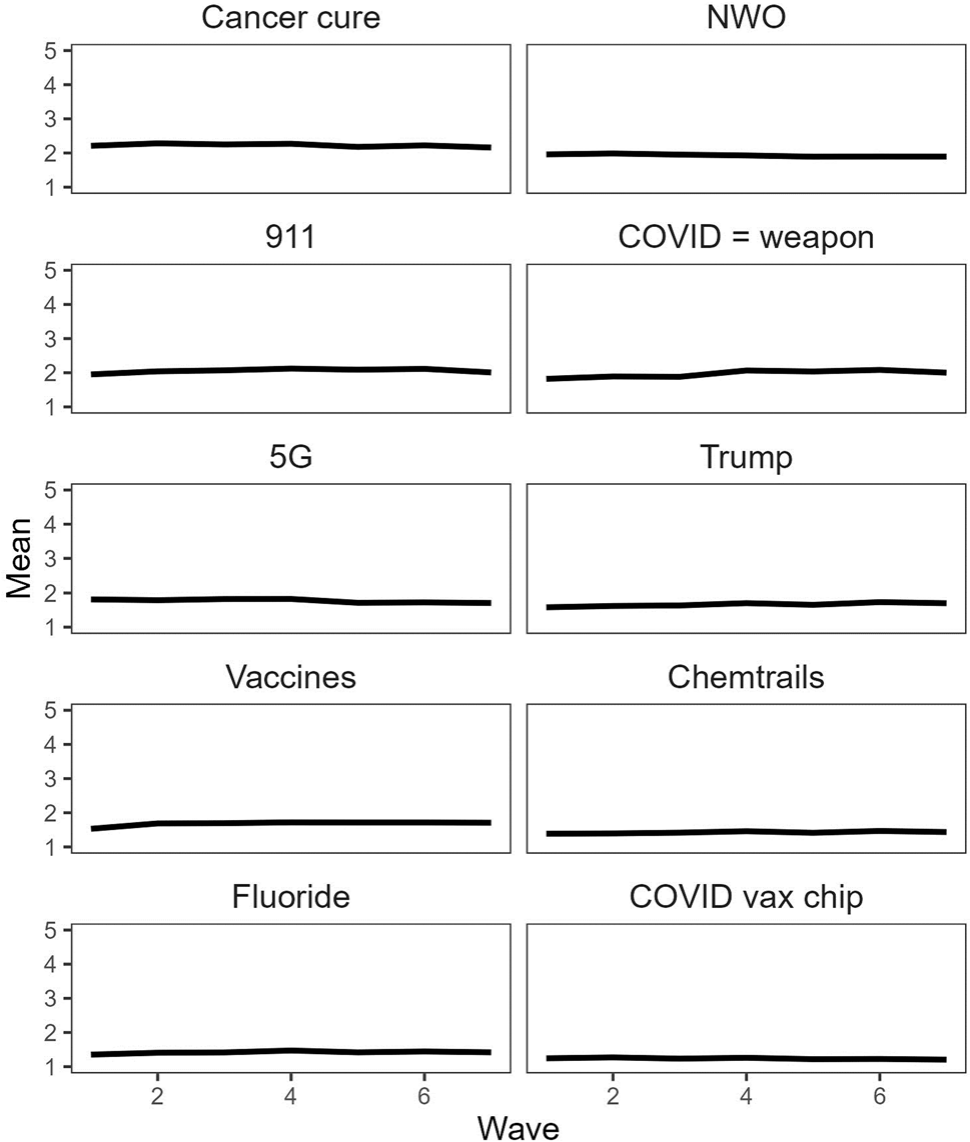
Changes in mean agreement with conspiracy theories over time.

In our next analysis, we examined changes in beliefs over time at the level of the overall sample. The mean level of agreement with each conspiracy theory at each time point is displayed in Fig. 1. As is clear in the graph, there was relatively little change in average levels of conspiracy beliefs over time. A partial exception was the conspiracy theory that COVID-19 is a biological weapon intentionally created and released by China, for which there was a bump in agreement levels of approximately 0.19 points between wave 3 in May and wave 4 in June.

To check whether there was any evidence of an upward trend in beliefs over time, we estimated a mixed model where an individual’s response to an individual item at a given point in time was the outcome variable, and wave (1–7) was the predictor variable, with an assumed linear effect. This model included random intercepts across items and participants. This indicated no evidence of a linear trend in beliefs in conspiracy theories: The effect of time on responses (per month) was miniscule, *B* = 0.003, 95% CI [− 0.001, 0.008].

### Within-person standard deviations of scores

While the analysis above provides some information about how beliefs in conspiracy theories fluctuated in the sample as a whole, it does not convey the degree to which *individual* participants’ beliefs in conspiracy theories changed over time. We therefore calculated each participants’ conspiracy theory score at each wave (as the mean of their responses to the ten items at each wave), and then calculated the within-person standard deviation of these scores across all the waves they took part in.

Considering the possible range of 1 to 5 for conspiracy theory scores, the within-person standard deviations were quite small, with a mean of 0.16 and an interquartile range of 0.08 to 0.21. Comparing these estimates to those reported above, it is clear that there was much less within-person variance than between-person variance. Participants who had higher levels of belief (averaged across all items and waves) tended to have higher within-person standard deviations, Spearman’s *r* = 0.62, *p* < 0.001.

### Trajectories of change and stability

The previous subsection provides some indication of the incidence of substantial changes in beliefs over the course of the study, but does not illustrate the specific trajectories displayed by individual participants. For the purposes of succinctly describing different trajectories of belief over the study period, we provide the following classifications and descriptive labels of individual change/stability (Table 3). We acknowledge that there are alternative thresholds and approaches that could be used and encourage researchers to adapt our analysis code to further explore our open data.

**Table 3:** Trajectory definitions.

Trajectory	Definition
Consistent sceptic	The participant strongly disagreed (1), somewhat disagreed (2) or neither agreed nor disagreed (3) with the theory at every wave for which they provided a response
Bump	The participant began the study either disagreeing with the theory or being neutral towards it (0–3), then somewhat (4) or strongly agreed (5) with the theory at least once during waves two to six, but returned to disagreement or neutrality (0–3) by wave seven
Convert	The participant began the study either disagreeing with the theory or being neutral towards it (responses 0–3), but later shifted to somewhat (4) or strongly (5) agreeing with the theory, sustaining agreement at wave seven
Apostate	The participant began the study somewhat or strongly agreeing with the theory at wave one, but later shifted to disagreement or neutrality, and had not returned to agreeing with the theory at wave seven
Dip	The participant began the study somewhat or strongly agreeing with the theory, then indicated disagreement or neutrality at least once during waves two to six, but returned to agreement by wave seven
Consistent believer	The participant indicated agreement (4 or 5) throughout

These trajectories apply to each participant’s responses to an individual conspiracy theory; participants could have different trajectories for different theories.

**Figure 2:**
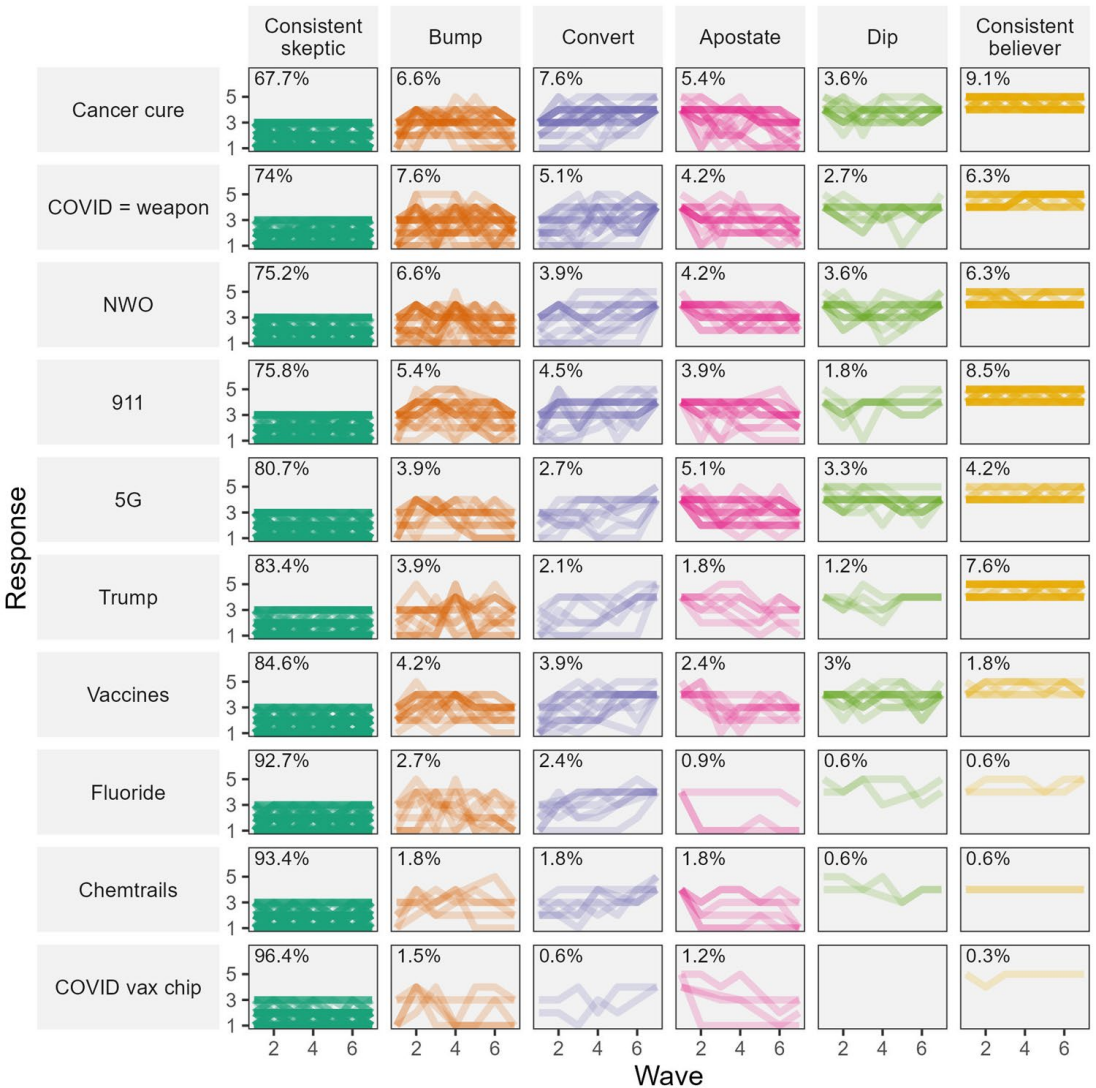
Plot of trajectories over time. Panels display percentage of participants in each trajectory group (columns), for each item (rows). Plotted lines represent agreement with item over time for individual participants.

We only included participants with complete data at waves 1 and 7 in this analysis but allowed missing data for the intervening waves. A plot of trajectories is displayed in Fig. 2. “Consistent sceptic” trajectories were by far the most common, representing well over half of the trajectories for each theory. Nevertheless, a variety of trajectories were apparent in the data, included a small minority of convert and apostate trajectories for each theory (these trajectories involving substantial and unreversed changes in belief). That said, the percentage of participants in the convert and apostate trajectories tended to be quite similar for each theory. That is, although each theory attracted new converts during the data collection period, it also lost a similar number of apostates.

### Intraclass correlation coefficient

While the analyses presented above provide detailed information about within-person variation in beliefs in conspiracy theories, they do not summarise this information in a simple metric that facilitates comparisons across studies. A useful such metric is the intraclass correlation coefficient (ICC). We calculated the ICC using participant’s overall scores (i.e., averaged across all ten items) at each of the seven time points. This aggregation across items implicitly excludes variation due to different theories from this analysis. The intraclass correlation was calculated using the ICC package^25^ in R, with a confidence interval calculated using the “THD” exact method. The resulting ICC was *r* = 0.91, 95% CI [0.90, 0.92]. In other words, approximately 91% of the variation in conspiracy scores over time was accounted for by between-person differences.

### Accounting for measurement error

The analyses presented above provide an indication of the degree to which responses varied over time within participants, and the degree to which variation in responses can be attributed to stable individual differences. However, at least some of the variation in responses over time within participants will inevitably have been caused by measurement error. In principle, it is impossible to determine with certainty the degree to which any given response was affected by measurement error. However, by adding assumptions it is possible to specify a statistical model that separates measurement error from “true” changes in beliefs.

To achieve this, we specified a multilevel structural equation model with one between-person latent variable and one within-person latent variable, each loading on all items. In other words, responses to items were assumed to be accounted for by unidimensional models at both the inter- and within-person levels. Encapsulated in this model is the assumption that observed within-person variation in responses is produced either by measurement error unique to the person, item, and time, or by variation in an underlying unidimensional “conspiracism” latent variable that affects a participant’s responses to *all* the items at a given time point.

We estimated the model via maximum likelihood estimation with robust Huber-White standard errors and scaled test statistics to address non-normality (“MLR” estimation) in lavaan^26^. This model permitted us to estimate the standard deviation of a within-person “conspiracism” latent variable having effects on responses all items for a person at a given time point. The model fitted the covariance matrix reasonably well, albeit imperfectly. The robust root mean square error of approximation of 0.043 was within Hu and Bentler’s^27^ cut-off of 0.06 for good fit, although the scaled chi-square test indicated a null hypothesis of perfect fit in the population could be rejected, χ^2^(70) = 270, *p* < 0.001.

In this model, the estimated variance of the between-person conspiracism latent variable was 0.515, 95% CI [0.392, 0.638]. Since we used the marker variable approach to scale the latent variables, this variance is set on the same 1–5 scale as the original item responses, and suggests substantial between-person variance. The estimated variance of the within-person latent variable was just 0.016, 95% CI [0.001, 0.030]. This suggested the presence of very little within-person variance after measurement error had been accounted for. Full parameter estimates for this model can be found in Supplementary Table 3.

## Discussion

A small number of participants substantially revised their beliefs about a conspiracy theory during our study. Belief (or disbelief) in conspiracy theories is thus clearly not immutable. Yet the overall pattern observed in our study was very much one of stability, with much less variance in beliefs within persons than between persons. Our results cohere with those of previous longitudinal studies in which beliefs in conspiracy theories were relatively stable over time^4–6,11^. People developing beliefs in a succession of conspiracy theories are often characterised as falling down a “rabbit hole”—a reference to Lewis Carroll’s *Alice’s Adventures in Wonderland*^28^. It seems that if conspiracy theorists do fall down a rabbit hole, it is typically one with a rather gradual slope.

Despite popular concerns about a contemporary misinformation “infodemic”^29–31^, we found no evidence of a general increase in beliefs in conspiracy theories over the course of our study. On average, beliefs in conspiracy theories remained relatively low and stable over the course of the study, with no evidence of any significant linear trend. This was despite our data collection occurring during a time of societal turmoil—the second year of the COVID-19 pandemic, when COVID-19 vaccinations were just beginning in Australia and New Zealand, and lockdowns were still being implemented for some periods and locations^14^. Although we did not construct our study to be a test of any particular theory, the lack of an increase in beliefs in conspiracy theories is difficult to reconcile with explanatory accounts suggesting that distressing societal events provoke belief in conspiracy theories (e.g., the existential threat model^32^).

Our findings in this regard cohere with recent investigations of long-term trends in conspiracy theory beliefs^33,34^. A notable finding of our within-person trajectories over time was that individuals who began agreeing with a specific conspiracy theory but later became neutral or disagreed (i.e., apostate), were more or less offset by an equal number of individuals who began disagreeing or neutral towards a specific conspiracy theory but later indicated agreement with it (i.e., convert). This may help explain a lack of evidence for an average increase in beliefs in conspiracy theories over time, consistent with past research^33,34^, alongside growing concerns of a visible increase in conspiracy theories^35^.

### Theoretical implications

A variety of exogeneous factors or “motives” have been invoked to explain beliefs in conspiracy theories. Douglas et al.^2^ categorise these motives as epistemic (e.g., tendency toward intuitive rather than analytic thinking), existential (e.g., powerlessness, anxiety, alienation), and social (e.g., collective narcissism). All of these factors vary to at least some extent between and within people. The challenge for theorists posed by our findings is to explain why variance in such factors or motives is sufficient to produce substantial between-person variance in beliefs in conspiracy theories, but *not* sufficient to produce more than a very small quantity of within-person variance in studies such as ours. More broadly, whatever theories or models are invoked to explain belief in conspiracy theories must accommodate the observation that such beliefs appear to be very stable.

### Methodological implications

From a methodological perspective, our findings signal a caution for researchers interested in using longitudinal research to estimate the causes or consequences of within-person variation in beliefs in conspiracy theories. A popular analytic method for addressing such topics is the RI-CLPM^36^. Analyses using the RI-CLPM are typically intended to draw inferences about variation that is neither due to stable individual differences (which are partialled out of the analysis) nor due to measurement error. However, our findings imply that stable individual differences and measurement error are the *predominant* causes of variation in responses in a longitudinal dataset such as ours, with true within-person changes in belief playing a much smaller role.

Power analyses we conducted with the powRICLPM package^37^ suggest that the stability of beliefs in conspiracy theories in our study imply that longitudinal studies using the RI-CLPM need large samples of participants and time points to achieve adequate statistical power (see the Supplementary Information). For example, with four time points, even a sample of 1000 with no missing data whatsoever may be insufficient to achieve 80% power to detect a cross-lagged effect of standardised *b* = 0.2. We strongly encourage researchers planning longitudinal studies to conduct power analyses that are informed by realistic assumptions regarding temporal stability.

### Limitations

Our study used convenience sampling, and our participants tended to be relatively highly educated, liberal, and young (see Supplementary Table 1). Our findings thus may not generalise to the wider population of residents of Australia and New Zealand (nor to populations of other countries). Relatively few of our participants displayed a high level of agreement with multiple conspiracy theories. While this is a typical finding of research in this area, it does mean that our findings may not generalise to samples or populations with very high levels of belief in conspiracies.

The small proportion of politically conservative participants in our sample (9%) similarly implies that our findings might not necessarily generalise to conservative populations. Furthermore, because conservatism was correlated with higher levels of belief in the conspiracy theories in our sample, and higher levels of belief in conspiracy theories was correlated with more within-person variance, it is possible that the low representation of conservatives might have biased our estimates of within-person variance downward in some analyses. That said, a robustness analysis where we estimated the ICC within the liberal, moderate, and conservative subsamples produced very similar estimates in each subsample, with very narrow confidence intervals (see the Supplementary Information). This suggests that a sample that was more representative of the general Australasian population in terms of distribution of political ideology would be unlikely to have substantially altered our estimated ICC.

While we conducted a structured evaluation process to enhance the content validity of our items (see the Supplementary Information), and our multilevel SEM analysis provides some tentative evidence of factorial validity, our measure of belief in conspiracy theories was not rigorously validated prior to the study. Replications of our finding using alternative measures may be useful^see^^38^.

Our items related to a small number of conspiracy theories that were *contemporary* but nevertheless disseminated and popularised prior to our study. We thus cannot make inferences about how participants react to *new* conspiracy theories in the period immediately following their introduction into popular discourse. It is plausible that much more within-person change might be observed in such periods. Relatedly, our finding of stability in beliefs of participant might not hold in cases where epistemic authorities publicly revise their conclusions about a specific conspiracy theory. More broadly, we cannot assume that our findings would generalise to the wider class of all conspiracy theories. In addition, we could only measure participants’ expressed *agreement* with theories, not whether or not these expressions reflected deeply held beliefs^see^^39^.

The conspiracy theories we included in our survey were *specific* theories; we did not include any measures of a broader conspiracy mentality. We were thus unable to test Imhoff et al.’s^9^ hypothesis that conspiracy mentality is more stable than beliefs in specific theories. This hypothesis could be tested in future longitudinal studies.

A final limitation pertains to our use of structural equation modelling to differentiate true within-person variation in beliefs from measurement error. The model we specified assumes that within-person variation in item responses is explained by a unidimensional factor model. Although this model fitted the data reasonably well, it may still not be the correct model. Indeed, recent research has suggested that—at least in cross-sectional data—a unidimensional model does not adequately explain relationships between beliefs in conspiracy theories^40^. The potential of model misspecification implies the existence of more uncertainty around the estimated within-person variance than is indicated in its confidence interval.

### Supplementary Information

Supplementary Information.

## Data Availability

The data, analysis code and materials that support the findings of this study are openly available on the Open Science Framework at https://osf.io/t7upg/.
