# Supplementary material for: People do change their beliefs about conspiracy theories—but not often
Source: Sci Rep. 2024 Feb 15;14:3836. doi: 10.1038/s41598-024-51653-z (PMC10869812; doi:10.1038/s41598-024-51653-z)
Supplement: Supplementary file 1 — Supplementary Information. [file 41598_2024_51653_MOESM1_ESM.docx]

Supplementary information for manuscript
“People do change their beliefs about conspiracy theories—but not often”

Contents

[Precision Analysis for Sample Size 2](#_Toc152943489)

[Additional Demographic Information 2](#_Toc152943490)

[Prolific Payments 3](#_Toc152943491)

[Measure Construction 4](#_Toc152943492)

[Ranges of Beliefs Within Participants and Theories 5](#_Toc152943493)

[Correlations Across Time Points 6](#_Toc152943494)

[Parameter Estimates for Multilevel Structural Equation Model (SEM) 7](#_Toc152943495)

[Alternative Random-Intercept Lagged Panel Model 9](#_Toc152943496)

[Parameter Estimates 11](#_Toc152943497)

[Implications for Power Analyses 16](#_Toc152943498)

[Political Ideology and Belief in Conspiracy Theories 18](#_Toc152943499)

[Within-Person Standard Deviations by Political Ideology 19](#_Toc152943500)

[Intraclass Correlation Coefficient (ICC) by Political Ideology 20](#_Toc152943501)

[Implications for Credibility of Study 21](#_Toc152943502)

[Beliefs in Warranted Conspiracy Theories 21](#_Toc152943503)

[Between-person Variation 22](#_Toc152943504)

[Change in Mean Agreement Over Time 22](#_Toc152943505)

[Ranges of Belief Within Participants and Theories 23](#_Toc152943506)

[Trajectories of Change and Stability 24](#_Toc152943507)

[Intraclass Correlation Coefficient (ICC) 25](#_Toc152943508)

[Accounting for Measurement Error 25](#_Toc152943509)

[Correlation between beliefs in Warranted and Unfounded Conspiracy Theories 25](#_Toc152943510)

[Predictors of Completing More Waves 25](#_Toc152943511)

[Time 1 Survey Data 26](#_Toc152943512)

[Prolific Participation Variables 29](#_Toc152943513)

[Statistical Assumptions of Analyses Presented in Main Text 31](#_Toc152943514)

[References 34](#_Toc152943515)

# Precision Analysis for Sample Size

This study does not involve tests of hypotheses or an emphasis on significance testing, meaning that statistical power analysis was not a suitable tool for sample size determination. Instead, the target sample size (approximately *N* = 500) was determined by practical and financial constraints. We nevertheless conducted a precision analysis for the intraclass correlation coefficient (a key output of our study) using formula 5 in Zou^1^. We made the relatively conservative assumption that the true ICC was 0.6 or higher (the closer to 0.5, the wider the confidence interval). This precision analysis suggested that with 360 participants each answering four or more of the seven time points, the probability that the 95% confidence interval for the ICC would be less than 0.10 is greater than 95% (i.e., assurance probability of 95%). This indicates that the sample size achieved in this study (see below) is more than sufficient to estimate the ICC precisely.

# Additional Demographic Information

Supplementary Table 1
*Demographic Characteristics of Participants (time 1)*

|  | Australia  (*n* = 401) | | New Zealand  (*n* = 97) | |
| --- | --- | --- | --- | --- |
|  | *n* | % | *n* | % |
| Gender |  |  |  |  |
| Male | 211 | 52.6 | 50 | 51.5 |
| Female | 186 | 46.4 | 47 | 48.5 |
| Non-binary / gender diverse | 4 | 1 | 0 | 0 |
| Highest level of completed education |  |  |  |  |
| Doctoral degree (e.g., PhD, PsyD, MD) | 22 | 5.5 | 1 | 1 |
| Postgraduate degree (e.g., Masterate) | 68 | 17.0 | 15 | 15.5 |
| Undergraduate degree (e.g., Bachelor's) | 179 | 44.6 | 45 | 46.4 |
| Other tertiary qualification | 62 | 15.5 | 13 | 13.4 |
| Completed high school | 63 | 15.7 | 22 | 22.7 |
| Some high school (without completing) | 7 | 1.7 | 1 | 1 |
| No high school | 0 | 0 | 0 | 0 |
| Political orientation |  |  |  |  |
| Liberal (\left-wing) | 183 | 45.6 | 44 | 45.4 |
| Moderate | 169 | 42.1 | 40 | 41.2 |
| Conservative (\right-wing) | 36 | 9 | 8 | 8.2 |
| Other | 13 | 3.2 | 5 | 5.2 |
| Personal income before tax per annum* |  |  |  |  |
| Less than $20,000 | 105 | 26.2 | 34 | 35.1 |
| $20,000 - $39,999 | 74 | 18.5 | 17 | 17.5 |
| $40,000 - $59,999 | 62 | 15.5 | 20 | 20.6 |
| $60,000 - $79,999 | 59 | 14.7 | 11 | 11.3 |
| $80,000 - $99,999 | 49 | 12.2 | 4 | 4.1 |
| $100,000 or more | 52 | 13 | 11 | 11.3 |
| Employment status |  |  |  |  |
| Working full-time | 172 | 42.9 | 42 | 43.3 |
| Working part-time | 93 | 23.2 | 22 | 22.7 |
| Unemployed and looking for work | 34 | 8.5 | 9 | 9.3 |
| A homemaker or stay-at-home parent | 17 | 4.2 | 5 | 5.2 |
| Student | 62 | 15.5 | 16 | 16.5 |
| Retired | 8 | 2 | 0 | 0 |
| Other (employment) | 15 | 3.7 | 3 | 3.1 |
| Marital status |  |  |  |  |
| Married | 118 | 29.4 | 27 | 27.8 |
| Living with a partner | 76 | 19 | 16 | 16.5 |
| Widowed | 1 | 0.2 | 0 | 0 |
| Divorced/separated | 18 | 4.5 | 2 | 2.1 |
| Never married | 188 | 46.9 | 52 | 53.6 |

*Notes*. Income is displayed in Australian dollars for Australian participants and New Zealand dollars for New Zealand participants.

# Prolific Payments

Almost all submissions from participants were approved for payment, including those who failed attention checks. The only exceptions were two occasions where a participant both failed an attention check *and* answered a survey extremely quickly (i.e., well under half the median duration).

At time 5, the lead researcher inadvertently scheduled the survey to be advertised on Prolific while it was paused for submissions on Prolific. This meant that participants entering the survey via Prolific could not open it, and many timed-out, returned and “NOCODE” submissions were received. All participants who attempted the inactive survey were paid (regardless of their submission status) and sent an apology message. A corrected link was then re-distributed on Prolific on the same day. This meant that many participants were paid twice at time 5.

# Measure Construction

Beyond a selection of demographic items, the only measure of interest in the current study was our set of conspiracy theory items. We chose not to use a pre-existing measure of belief in conspiracy theories such as the Belief in Conspiracy Theories Inventory^2^ to ensure that the conspiracy theories presented in our items were of contemporary relevance. This increased the plausibility that we might see fluctuations in belief over time. We constructed and adapted items to fulfil the following requirements:

1. Each item must describe a conspiracy theory—i.e., a claim involving the elements of *multiple* actors plotting together, *secrecy,* and *malevolence* (i.e., harmful goals). Although some definitions of “conspiracy theory” involve additional elements ^see 3^, these three core elements are common to many definitions of conspiracy theory ^e.g., 4–6^. This criterion was specified to safeguard the content validity of items.
2. The theory described in each item must be *notable—*i.e., it must have been covered in Wikipedia and/or mainstream media articles. This avoided including very obscure theories which might be unfamiliar to most participants.
3. The theory described in each item must relate to claimed events which are either ongoing or occurred in the last 20 years (at the time of data collection). This helped to ensure contemporary relevance.
4. The item must be clear and easy to read.

Items were reviewed and revised via a structured review process until each member of the project team was satisfied that each of these conditions were met for each item included (with two deliberate exceptions; see below). The final list of items and their sources can be found in Table 2 of the main manuscript.

# Ranges of Beliefs Within Participants and Theories

In addition to the analyses of within-person change reported in the main text, we also conducted an analysis of the range of beliefs within participants and theories. For every participant and conspiracy theory we calculated the range (maximum - minimum) of responses over time. This provided a simple indication of the magnitude of within-person variation. Only the 472 participants who responded to at least two waves were included in this analysis.

Fifty-three percent of the ranges thus calculated were zero. In other words, if one selects a conspiracy theory and participant from this sample of 472 participants at random, there is a greater than even chance that the participant indicated exactly the same level of agreement with the conspiracy theory at every wave they responded to. However, for 16% of the combinations of participant and conspiracy theory, the participant’s highest level of agreement was at least two points greater than their lowest. Two points is a relatively substantial difference in this context, indicating a change from strong [dis]agreement (1) to neither agree nor disagree (3), or from agreement (4) to disagreement (2), or vice versa. A bar plot demonstrating the frequency of ranges is presented in Supplementary Figure 1.

Supplementary Figure 1
*Proportion of Belief Changes Across Beliefs*


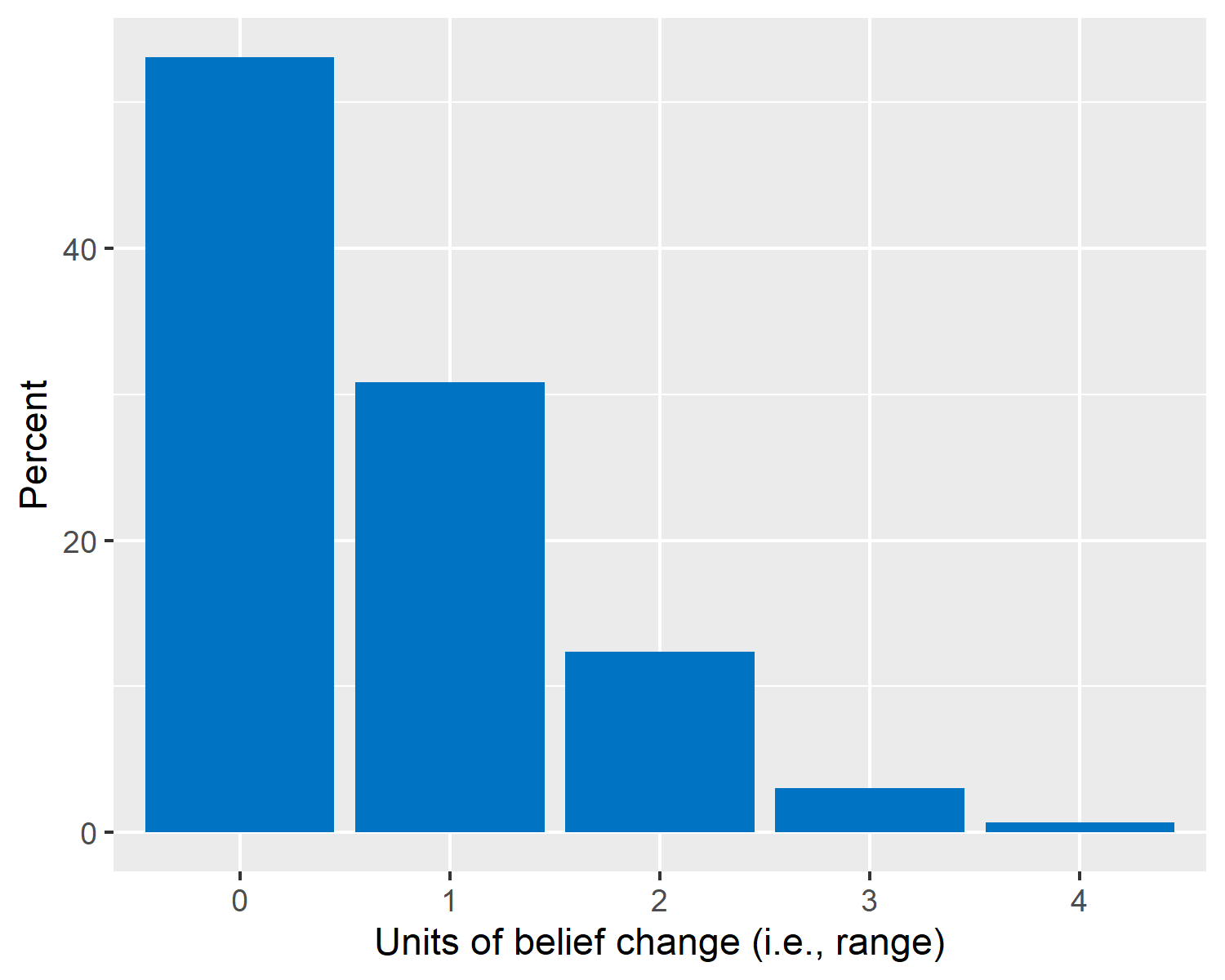


*Note*. For each of the 472 participants who responded to at least two waves, a range (max-min) was calculated for their responses to each of the ten theories. Excluding missing data points, there are thus 4719 ranges plotted in this frequency distribution.

# Correlations Across Time Points

For comparison with other studies, it is useful to report a Pearson’s correlation matrix between scores on the measure of conspiracy theories at different time points. This is presented in Supplementary Table 2.

Supplementary Table 2
*Pearson’s Correlations Between Conspiracy Scores Across Times*

|  | Time 1 | Time 2 | Time 3 | Time 4 | Time 5 | Time 6 | Time 7 |
| --- | --- | --- | --- | --- | --- | --- | --- |
| Time 1 | 1.00 |  |  |  |  |  |  |
| Time 2 | .92 | 1.00 |  |  |  |  |  |
| Time 3 | .91 | .94 | 1.00 |  |  |  |  |
| Time 4 | .88 | .92 | .93 | 1.00 |  |  |  |
| Time 5 | .89 | .90 | .92 | .94 | 1.00 |  |  |
| Time 6 | .88 | .90 | .92 | .91 | .96 | 1.00 |  |
| Time 7 | .86 | .88 | .91 | .91 | .95 | .96 | 1.00 |

*Notes*. Pairwise complete observations used to compute each coefficient.

# Parameter Estimates for Multilevel Structural Equation Model (SEM)

In our main text, we briefly report estimates from a multilevel SEM, allowing us to estimate the quantity of within-person variance in an underlying conspiracism latent variable (after accounting for measurement error). For space reasons, we did not report full parameter estimates for this model in the main text. They can be found below in Supplementary Table 3.

Supplementary Table 3
*Parameter Estimates for Multilevel Structural Equation Model*

|  |  | 95% CI | |
| --- | --- | --- | --- |
|  | Est.^1^ | Lower | Upper |
| **Within-person level** |  |  |  |
| Factor loadings |  |  |  |
| consp_w =~ consp_covidweap | 1.000 | 1.000 | 1.000 |
| consp_w =~ consp_NWO | 1.071 | 0.591 | 1.551 |
| consp_w =~ consp_5G | 1.164 | 0.667 | 1.661 |
| consp_w =~ consp_covidvax | 1.131 | 0.398 | 1.864 |
| consp_w =~ consp_chemtrails | 1.496 | 0.732 | 2.259 |
| consp_w =~ consp_fluor | 1.583 | 0.620 | 2.546 |
| consp_w =~ consp_911 | 1.339 | 0.751 | 1.927 |
| consp_w =~ consp_vaxx | 1.463 | 0.771 | 2.154 |
| consp_w =~ consp_Trump | 1.345 | 0.741 | 1.949 |
| consp_w =~ consp_cancercure | 1.319 | 0.721 | 1.917 |
| Error variances |  |  |  |
| consp_covidweap ~~ consp_covidweap | 0.305 | 0.262 | 0.348 |
| consp_NWO ~~ consp_NWO | 0.257 | 0.225 | 0.290 |
| consp_5G ~~ consp_5G | 0.185 | 0.158 | 0.211 |
| consp_covidvax ~~ consp_covidvax | 0.086 | 0.067 | 0.105 |
| consp_chemtrails ~~ consp_chemtrails | 0.129 | 0.104 | 0.154 |
| consp_fluor ~~ consp_fluor | 0.140 | 0.112 | 0.168 |
| consp_911 ~~ consp_911 | 0.223 | 0.191 | 0.254 |
| consp_vaxx ~~ consp_vaxx | 0.191 | 0.167 | 0.215 |
| consp_Trump ~~ consp_Trump | 0.158 | 0.128 | 0.188 |
| consp_cancercure ~~ consp_cancercure | 0.270 | 0.235 | 0.305 |
| Factor variance |  |  |  |
| consp_w ~~ consp_w | 0.016 | 0.001 | 0.030 |
|  |  |  |  |
| **Between-person level** |  |  |  |
| Factor loadings |  |  |  |
| consp_b =~ consp_covidweap | 1.000 | 1.000 | 1.000 |
| consp_b =~ consp_NWO | 1.155 | 1.009 | 1.302 |
| consp_b =~ consp_5G | 1.097 | 0.928 | 1.265 |
| consp_b =~ consp_covidvax | 0.585 | 0.459 | 0.710 |
| consp_b =~ consp_chemtrails | 0.799 | 0.662 | 0.935 |
| consp_b =~ consp_fluor | 0.841 | 0.686 | 0.997 |
| consp_b =~ consp_911 | 1.165 | 0.994 | 1.336 |
| consp_b =~ consp_vaxx | 0.903 | 0.759 | 1.048 |
| consp_b =~ consp_Trump | 0.960 | 0.800 | 1.120 |
| consp_b =~ consp_cancercure | 1.157 | 1.021 | 1.294 |
| Error variances |  |  |  |
| consp_covidweap ~~ consp_covidweap | 0.489 | 0.374 | 0.603 |
| consp_NWO ~~ consp_NWO | 0.308 | 0.227 | 0.388 |
| consp_5G ~~ consp_5G | 0.363 | 0.273 | 0.452 |
| consp_covidvax ~~ consp_covidvax | 0.098 | 0.064 | 0.133 |
| consp_chemtrails ~~ consp_chemtrails | 0.114 | 0.080 | 0.148 |
| consp_fluor ~~ consp_fluor | 0.132 | 0.088 | 0.176 |
| consp_911 ~~ consp_911 | 0.593 | 0.476 | 0.710 |
| consp_vaxx ~~ consp_vaxx | 0.267 | 0.202 | 0.332 |
| consp_Trump ~~ consp_Trump | 0.602 | 0.455 | 0.749 |
| consp_cancercure ~~ consp_cancercure | 0.577 | 0.474 | 0.681 |
| Factor variances |  |  |  |
| consp_b ~~ consp_b | 0.515 | 0.392 | 0.638 |
| Intercepts^2^ |  |  |  |
| consp_covidweap ~1 | 1.915 | 1.825 | 2.006 |
| consp_NWO ~1 | 1.904 | 1.814 | 1.994 |
| consp_5G ~1 | 1.755 | 1.666 | 1.844 |
| consp_covidvax ~1 | 1.237 | 1.189 | 1.284 |
| consp_chemtrails ~1 | 1.405 | 1.345 | 1.466 |
| consp_fluor ~1 | 1.405 | 1.341 | 1.469 |
| consp_911 ~1 | 2.031 | 1.929 | 2.133 |
| consp_vaxx ~1 | 1.636 | 1.561 | 1.711 |
| consp_Trump ~1 | 1.632 | 1.539 | 1.724 |
| consp_cancercure ~1 | 2.212 | 2.111 | 2.314 |
| consp_b ~1 | 0.000 | 0.000 | 0.000 |

*Notes*. ^1^Unstandardised estimates. ^2^Intercepts are by default fixed to 0 at the within-person level.

# Alternative Random-Intercept Lagged Panel Model

The SEM model reported in the main text was not the first model we developed for the purpose of addressing measurement error, but rather one we discovered during the peer review process for an earlier version of this manuscript. Initially (e.g., in our first preprint), we specified a structural equation model inspired by the multiple indicator random intercept cross-lagged panel model (RI-CLPM) of Mulder and Hamaker^7^ (Figure 3, top panel). This model excludes a cross-lagged component since we are measuring just one attribute (belief in conspiracy theories), and is thus a multiple indicator random-intercept lagged panel model (RI-LPM). We include it here as a robustness analysis, demonstrating that the estimate of within-person variance remains very similar with an alternative modelling approach.

The model uses responses to individual items across time points and participants as the input data, and models these responses as functions of three sets of latent variables:

- “Random intercept” latent variables for each of the ten items. Each of these latent variables have factor loadings (fixed to one) that lead from the latent variable to the seven occasions on which the given item was measured. These random intercepts represent stable individual differences in how participants respond to each of the items.
- Seventy measurement error terms (one for each of the ten items and seven waves). These account for variation that is *unique* to a specific item, time, and participant.
- Seven within-person latent variables (one for each wave). Each of these latent variables have estimated factor loadings leading to the ten items measured at each wave. These latent variables represent variation that is unique to the participant and wave, but shared across items. Each factor loading (and its intercept) was constrained to equality across waves (i.e., a “strong invariance” model). Each within-person latent variable was connected to the one for the next time point via an autoregressive parameter.

An illustrative path diagram of this model (simplified to fit on a single page) is provided in Supplementary Figure 2. Parameters were estimated via maximum likelihood.

Supplementary Figure 2
*Illustrative Path Diagram for RI-LPM Measurement Model (Simplified)*


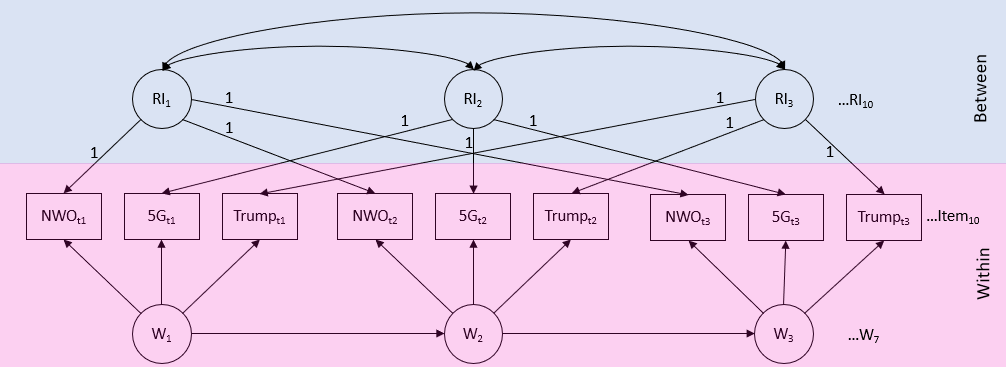


*Note*. This path diagram displays only three items and three time points, and is intended to convey the basic structure of the model specification. Displaying the path diagram of the full specified model (with ten items and seven time points) would be impractical on an A4 page. The oval “RI” terms represent random intercepts for items. The rectangular observed variables indicate responses to items. The oval “W” terms represent within-person variation over time.

Several ambiguities arose with respect to model specification and estimation for this RI-LPM, which is more complex than the multilevel SEM model reported in the main text. One of these ambiguities was with respect to whether the factor loadings and intercepts were fixed to equality across time points or permitted to vary over time. The model for which report parameter estimates below is a “strong” invariance model, where both the factor loadings and intercepts for the effects of the within-person factors were constrained to be fixed over the seven waves. It is also possible to specify models that assume only fixed loadings over time (“metric invariance”) or assume neither fixed intercepts nor fixed loadings over time (“configural invariance”).

When we attempted to estimate configural invariance and metric invariance models, they did converge, but produced “Heywood cases” – variance terms that were estimated to be negative (which is impossible). This may be because these models are overly complex relative to the small quantity of variation over time within participants, causing problems with identification. In contrast, the strong invariance model converged without errors or warnings. Although the purely descriptive goals of our study made the presence or absence of invariance over time a non-crucial issue, we report parameters for the strong invariance model below because of its relative simplicity, and because it permitted for tentative examination of whether the latent means varied over time. The absence of convincing evidence for longitudinal invariance of this model (and of its fit to the covariance matrix in general) mean that the parameter estimates from this model should be regarded as having additional uncertainty attached beyond that reflected in the standard errors and *p* values.

Another ambiguity in the SEM analysis related to the choice of estimator. lavaan offers [several](https://lavaan.ugent.be/tutorial/est.html) estimators. Of these, only a subset can be used with missing data present, which was crucial for our purposes given that many participants missed some waves. One appealing estimator was the diagonally weighted least squares (DWLS) estimator, which provides some robustness to non-normal and categorical data^8^, as was present in our study. However, DWLS estimation did not converge (neither for this model nor the multilevel SEM model reported in the main text).

We therefore instead used “MLR” estimation, which provides maximum likelihood estimation with robust Huber-White standard errors and scaled test statistics to address non-normality. This was also the estimator we used for the multilevel SEM reported in the main text. MLR estimation converged without warnings for the strong invariance RI-LPM.

## Parameter Estimates

Full parameter estimates for the RI-LPM can be found in Supplementary Table 4 and Supplementary Table 5. For current purposes, the most important parameter estimates are the variance terms. The ten random intercept variances were substantial relative to the 1-5 response scale; the mean of these variances was 0.80. The mean of the 70 measurement error variances was smaller, but still substantial, at 0.19. In contrast, the variances of the seven within-person latent variables were much smaller, with a mean of 0.02. Only one of these within-person variances was statistically significant (i.e., having a 95% confidence interval not including zero). As such, given the assumptions of this model, we can conclude that responses were much more strongly determined by stable individual differences and measurement error than by variation in conspiracism over time. Importantly, the estimate of within-person variance is nearly identical (to 2 decimal points) to that produced by the simpler multilevel model. As such, given that the multilevel model seems to be less prone to convergence problems and is much simpler to describe, we decided to use that in the main text of the manuscript.

As was the case for the multilevel SEM model, this RI-LPM model fitted the covariance matrix reasonably well, albeit imperfectly. The robust root mean square error of approximation of .037 was under Hu and Bentler’s cut-off of .06 for good fit, and the standardised root mean square residual of .041 was likewise under Hu and Bentler’s cut-off of .08. On the other hand, the scaled comparative fit index of .948 fell just outside of Hu and Bentler’s ^9^ suggested cut-off of .95, and the scaled chi-square test indicated a null hypothesis of perfect fit in the population could be rejected, χ^2^(2,392) = 3,598, *p* < .001.

Supplementary Table 4
*Parameter Estimates for RI-LPM Model (excluding Covariances)*

|  |  | 95% CI | |
| --- | --- | --- | --- |
|  | Unstd. Est.^1^ | Lower | Upper |
| Factor loadings^2^ |  |  |  |
| WF[x] =~ covidweap | 1.000 | 1.000 | 1.000 |
| WF[x] =~ NWO | 0.905 | 0.538 | 1.271 |
| WF[x] =~ FiveG | 1.069 | 0.594 | 1.545 |
| WF[x] =~ covidvax | 0.897 | 0.211 | 1.582 |
| WF[x] =~ chemtrails | 1.212 | 0.574 | 1.851 |
| WF[x] =~ fluoride | 1.251 | 0.414 | 2.087 |
| WF[x] =~ Sep11 | 1.217 | 0.694 | 1.739 |
| WF[x] =~ Vaxx | 1.308 | 0.654 | 1.962 |
| WF[x] =~ Trump | 1.268 | 0.723 | 1.812 |
| WF[x] =~ Cancercure | 1.244 | 0.655 | 1.833 |
| Autoregressions |  |  |  |
| WF_2 ~ WF_1 | 0.822 | 0.311 | 1.334 |
| WF_3 ~ WF_2 | 0.745 | 0.519 | 0.971 |
| WF_4 ~ WF_3 | 1.035 | 0.549 | 1.521 |
| WF_5 ~ WF_4 | 0.562 | 0.334 | 0.790 |
| WF_6 ~ WF_5 | 1.014 | 0.712 | 1.317 |
| WF_7 ~ WF_6 | 1.096 | 0.838 | 1.355 |
| Intercepts: |  |  |  |
| .covidweap | 1.885 | 1.790 | 1.979 |
| .NWO | 1.875 | 1.784 | 1.967 |
| .FiveG | 1.716 | 1.626 | 1.805 |
| .covidvax | 1.209 | 1.161 | 1.257 |
| .chemtrails | 1.373 | 1.311 | 1.435 |
| .fluoride | 1.373 | 1.307 | 1.439 |
| .Sep11 | 2.000 | 1.895 | 2.105 |
| .Vaxx | 1.607 | 1.529 | 1.686 |
| .Trump | 1.597 | 1.501 | 1.693 |
| .Cancercure | 2.170 | 2.068 | 2.273 |
| Latent means |  |  |  |
| .WF_2 | 0.035 | 0.004 | 0.067 |
| .WF_3 | 0.003 | -0.017 | 0.022 |
| .WF_4 | 0.026 | -0.004 | 0.055 |
| .WF_5 | -0.006 | -0.027 | 0.015 |
| .WF_6 | 0.018 | -0.002 | 0.038 |
| .WF_7 | -0.017 | -0.039 | 0.006 |
| Measurement error variances | |  |  |
| .covidweap.1 | 0.286 | 0.224 | 0.348 |
| .covidweap.2 | 0.323 | 0.223 | 0.422 |
| .covidweap.3 | 0.243 | 0.174 | 0.311 |
| .covidweap.4 | 0.369 | 0.252 | 0.485 |
| .covidweap.5 | 0.310 | 0.208 | 0.411 |
| .covidweap.6 | 0.302 | 0.217 | 0.386 |
| .covidweap.7 | 0.274 | 0.182 | 0.366 |
| .NWO.1 | 0.328 | 0.262 | 0.393 |
| .NWO.2 | 0.274 | 0.214 | 0.334 |
| .NWO.3 | 0.231 | 0.164 | 0.298 |
| .NWO.4 | 0.287 | 0.186 | 0.388 |
| .NWO.5 | 0.219 | 0.153 | 0.286 |
| .NWO.6 | 0.220 | 0.155 | 0.285 |
| .NWO.7 | 0.233 | 0.157 | 0.309 |
| .FiveG.1 | 0.276 | 0.216 | 0.336 |
| .FiveG.2 | 0.197 | 0.142 | 0.253 |
| .FiveG.3 | 0.172 | 0.116 | 0.228 |
| .FiveG.4 | 0.148 | 0.105 | 0.191 |
| .FiveG.5 | 0.135 | 0.086 | 0.185 |
| .FiveG.6 | 0.179 | 0.121 | 0.236 |
| .FiveG.7 | 0.148 | 0.095 | 0.202 |
| .covidvax.1 | 0.111 | 0.064 | 0.159 |
| .covidvax.2 | 0.108 | 0.051 | 0.165 |
| .covidvax.3 | 0.088 | 0.046 | 0.129 |
| .covidvax.4 | 0.085 | 0.047 | 0.122 |
| .covidvax.5 | 0.078 | 0.044 | 0.111 |
| .covidvax.6 | 0.076 | 0.046 | 0.106 |
| .covidvax.7 | 0.064 | 0.035 | 0.093 |
| .chemtrails.1 | 0.166 | 0.109 | 0.223 |
| .chemtrails.2 | 0.130 | 0.089 | 0.171 |
| .chemtrails.3 | 0.116 | 0.071 | 0.162 |
| .chemtrails.4 | 0.115 | 0.079 | 0.150 |
| .chemtrails.5 | 0.108 | 0.074 | 0.142 |
| .chemtrails.6 | 0.140 | 0.090 | 0.190 |
| .chemtrails.7 | 0.145 | 0.086 | 0.204 |
| .flouride.1 | 0.175 | 0.122 | 0.228 |
| .flouride.2 | 0.145 | 0.101 | 0.188 |
| .flouride.3 | 0.118 | 0.078 | 0.158 |
| .flouride.4 | 0.167 | 0.073 | 0.261 |
| .flouride.5 | 0.127 | 0.088 | 0.167 |
| .flouride.6 | 0.151 | 0.098 | 0.203 |
| .flouride.7 | 0.127 | 0.075 | 0.178 |
| .Sep11.1 | 0.247 | 0.188 | 0.305 |
| .Sep11.2 | 0.217 | 0.164 | 0.270 |
| .Sep11.3 | 0.263 | 0.177 | 0.349 |
| .Sep11.4 | 0.189 | 0.123 | 0.255 |
| .Sep11.5 | 0.191 | 0.139 | 0.243 |
| .Sep11.6 | 0.220 | 0.157 | 0.283 |
| .Sep11.7 | 0.206 | 0.149 | 0.262 |
| .Vaxx.1 | 0.230 | 0.177 | 0.284 |
| .Vaxx.2 | 0.182 | 0.139 | 0.224 |
| .Vaxx.3 | 0.229 | 0.168 | 0.291 |
| .Vaxx.4 | 0.183 | 0.138 | 0.228 |
| .Vaxx.5 | 0.157 | 0.117 | 0.197 |
| .Vaxx.6 | 0.165 | 0.120 | 0.210 |
| .Vaxx.7 | 0.171 | 0.115 | 0.226 |
| .Trump.1 | 0.199 | 0.125 | 0.273 |
| .Trump.2 | 0.144 | 0.097 | 0.191 |
| .Trump.3 | 0.151 | 0.098 | 0.204 |
| .Trump.4 | 0.177 | 0.070 | 0.284 |
| .Trump.5 | 0.115 | 0.071 | 0.158 |
| .Trump.6 | 0.123 | 0.079 | 0.168 |
| .Trump.7 | 0.153 | 0.051 | 0.256 |
| .Cancercure.1 | 0.384 | 0.306 | 0.461 |
| .Cancercure.2 | 0.345 | 0.266 | 0.423 |
| .Cancercure.3 | 0.258 | 0.183 | 0.333 |
| .Cancercure.4 | 0.229 | 0.155 | 0.303 |
| .Cancercure.5 | 0.202 | 0.145 | 0.259 |
| .Cancercure.6 | 0.193 | 0.141 | 0.245 |
| .Cancercure.7 | 0.212 | 0.141 | 0.283 |
| Random intercept variances | |  |  |
| RI_covidweap | 0.959 | 0.807 | 1.111 |
| RI_NWO | 0.960 | 0.822 | 1.098 |
| RI_FiveG | 0.925 | 0.779 | 1.071 |
| RI_covidvax | 0.248 | 0.157 | 0.339 |
| RI_chemtrails | 0.397 | 0.295 | 0.499 |
| RI_fluoride | 0.452 | 0.318 | 0.585 |
| RI_Sep11 | 1.234 | 1.069 | 1.398 |
| RI_Vaxx | 0.624 | 0.506 | 0.743 |
| RI_Trump | 1.025 | 0.827 | 1.224 |
| RI_Cancercure | 1.199 | 1.053 | 1.345 |
| Variances of within-person latents | | | |
| WF_1 | 0.039 | -0.003 | 0.081 |
| .WF_2 | 0.033 | -0.004 | 0.069 |
| .WF_3 | 0.012 | -0.001 | 0.026 |
| .WF_4 | 0.027 | -0.001 | 0.056 |
| .WF_5 | 0.016 | -0.005 | 0.036 |
| .WF_6 | 0.010 | 0.001 | 0.020 |
| .WF_7 | 0.004 | -0.005 | 0.014 |

*Notes.* ^1^Unstd. Est. = unstandardised estimate. ^2^Factor loadings on within-person factors were constrained to equality across time points, and therefore displayed just once here. Factor loadings for items on random intercepts were all constrained to 1, and therefore not shown. Covariance terms are displayed separately below.

Supplementary Table 5
*Covariances between Random Intercepts in RI-LPM Structural Equation Model*

|  | 1 | 2 | 3 | 4 | 5 | 6 | 7 | 8 | 9 | 10 |
| --- | --- | --- | --- | --- | --- | --- | --- | --- | --- | --- |
| 1. RI_covidweap | 1.00 |  |  |  |  |  |  |  |  |  |
| 2. RI_NWO | .61 | 1.00 |  |  |  |  |  |  |  |  |
| 3. RI_FiveG | .54 | .63 | 1.00 |  |  |  |  |  |  |  |
| 4. RI_covidvax | .51 | .61 | .60 | 1.00 |  |  |  |  |  |  |
| 5. RI_chemtrails | .56 | .70 | .65 | .71 | 1.00 |  |  |  |  |  |
| 6. RI_fluoride | .52 | .67 | .63 | .73 | .79 | 1.00 |  |  |  |  |
| 7. RI_Sep11 | .53 | .68 | .50 | .50 | .60 | .62 | 1.00 |  |  |  |
| 8. RI_Vaxx | .54 | .57 | .74 | .60 | .59 | .61 | .48 | 1.00 |  |  |
| 9. RI_Trump | .60 | .59 | .48 | .53 | .49 | .44 | .46 | .55 | 1.00 |  |
| 10. RI_Cancercure | .53 | .66 | .60 | .46 | .54 | .58 | .60 | .58 | .42 | 1.00 |

*Notes*. Covariances between random intercepts and within-person factors were all constrained to zero, and therefore are not shown here.

# Implications for Power Analyses

Although our longitudinal study was purely descriptive, it provides information that may be useful for future studies using longitudinal designs to test hypotheses about the causes and consequences of belief in conspiracy theories. One important statistical model suitable for analysing data from such longitudinal designs is the RI-CLPM^10^. The RI-CLPM uses random intercept terms to account for stable individual differences in the investigated variables. The researcher can then use cross-lagged terms (e.g., the effect of X at time 1 on Y at time 2, while controlling for Y at time 1) to draw inferences about causal effects. While this approach is not as conclusive as a true experiment, it does establish temporal precedence, and implicitly controls for all stable individual difference confounds. This model has already been applied in research on conspiracy theories^11,12^.

Given that the RI-CLPM controls for stable individual differences and makes inferences about cross-lagged effects using only within-person variation, its statistical power can be affected by the quantity of within-person variation. To illustrate this, we use the “powRICLPM” package for power analysis of bivariate RI-CLPM models^13^. We assume that a study is focused on detecting a cross-lagged effect (i.e., of variable X at time 1 on variable Y at time 2), and we hypothesise a moderate target effect size (standardised *b* = 0.2). We assume small positive correlations between the random intercepts (*r* = .2) and within-person residuals (*r* = .2), and substantial autoregressive effects (standardised *b* = 0.7). We then examine how power varies as a function of sample size (from *N* = 200 to *N* = 1000), number of time points (from 3 to 8), and intraclass correlation coefficient (i.e., the proportion of between-person variation in scores over time). We conducted 100 simulations/replications for each condition.

The results of our power analysis are displayed in Supplementary Figure 3. The analysis displays the importance of number of time points: With only three time points (the minimum required to identify an RI-CLPM), it is very difficult to achieve adequate power even with a low ICC and large sample size. The influence of the ICC is also very apparent: With an ICC of .6, as little as 4 time points and 500 participants may be sufficient to achieve 80% power. On the other hand, with an ICC of .9, much more data is needed: With 4 time points even *N* = 1000 is not quite to achieve 80% power, and with 5 time points an *N* of approximately 700 is required. Notably, an ICC of .9 is approximately what we found in this study—although it would vary in future studies depending on the samples and measures used, and the within-person variability of the predictor variables.

Even these power analyses make optimistic assumptions: They assume multivariate normal data, an absence of measurement error, and no missing data. In reality, data pertaining to beliefs about conspiracy theories is typically strongly skewed, will be affected by measurement error, and missing data in longitudinal studies is inevitable.

A notable limitation is that these power analyses were based on a simple RI-CLPM model that does not explicitly account for multiple indicators and measurement error. The more sophisticated multiple indicator RI-CLPM^7^ may have different power characteristics, which could be examined in future simulation studies.

Our code for these power analysis simulations can be found in the file “Power analysis with CRAN version of powRICLPM.R” at <https://osf.io/eqakz>. Our results depend on our specifications/inputs, and we thus encourage readers to examine how power varies across alternative specifications.

Supplementary Figure 3
*Output from Power Analyses for RI-CLPM Models with 3 to 8 time points*


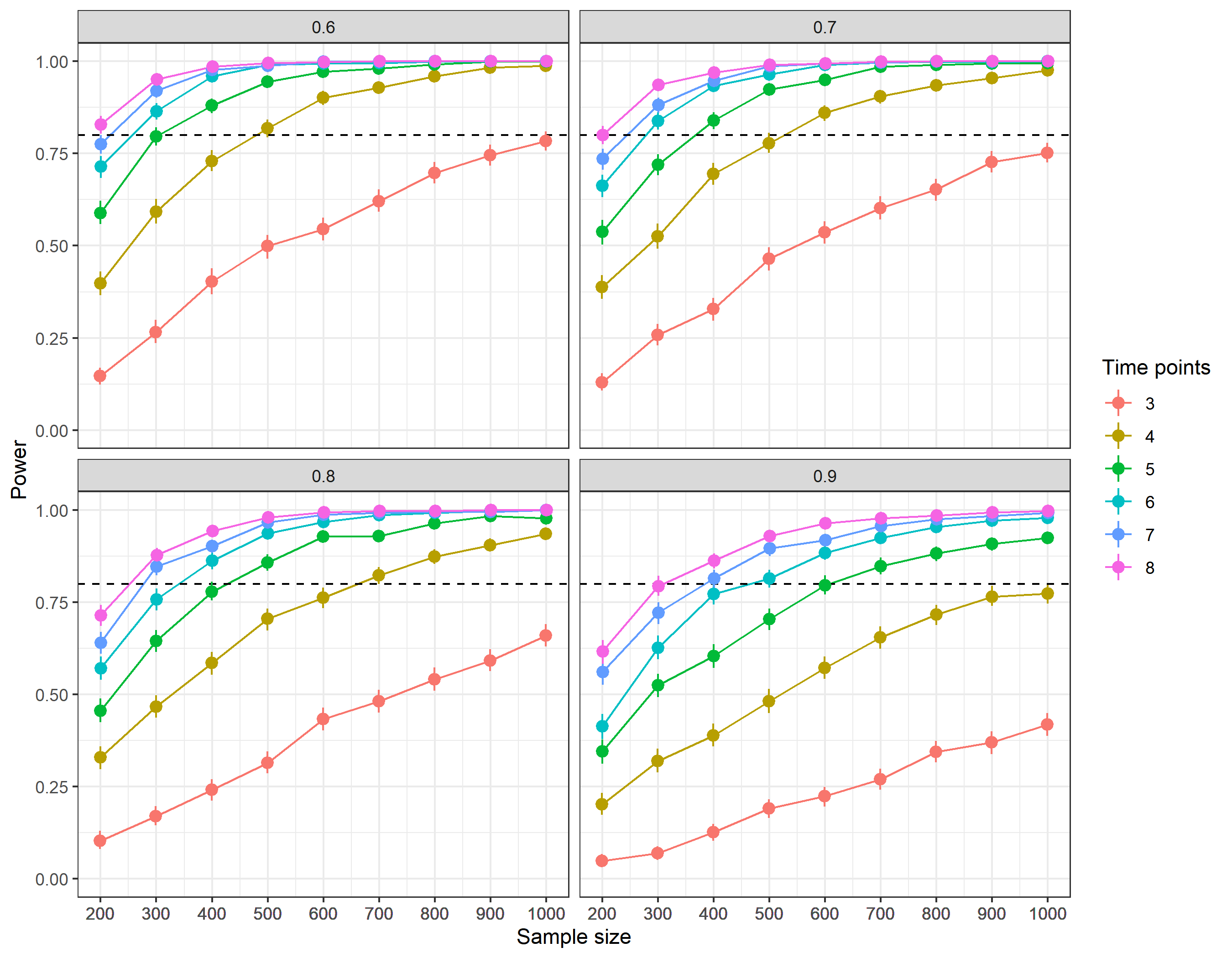


*Note*. The number at the top of each panel indicates the intraclass correlation (ICC).

# Political Ideology and Belief in Conspiracy Theories

Political ideology was not a major focus of our study. Nevertheless, some previous studies have reported a relationship between political ideology and belief in conspiracy theories. In particular, beliefs in conservative theories appear to be somewhat more common at ideological extremes, and especially amongst the extreme right^14^.

We therefore examined differences in mean conspiracy scores (averaged over all items and time points) across the four political ideology groups. This analysis suggested that conspiracy scores were higher in moderates and (especially) conservatives than in liberals. The small number of participants identifying as “other” had conspiracy theory scores similar to those of conservatives. A oneway ANOVA suggested that there was evidence of differences in means across groups, *F*(3, 494) = 32.53, *p* < .001. The standard deviations of scores were likewise higher for conservatives and moderates than for liberals.

Supplemental Table 6
*Beliefs in Conspiracy Theories by Political Ideology*

| Ideological group | *n* | *M* | *SD* |
| --- | --- | --- | --- |
| Liberal | 227 | 1.41 | 0.52 |
| Moderate | 209 | 1.88 | 0.71 |
| Conservative | 44 | 2.26 | 0.93 |
| Other | 18 | 2.17 | 0.94 |

*Notes*. A belief in conspiracy theories score was calculated for each participant by taking the mean of their responses to all items over all time points. Possible score range: 1 – 5.

This pattern was salient given that our sample was predominantly made up of participants who identified as liberal (45.6%) or moderate (42.0%) rather than conservative (8.8%) or other (3.6%). Although our political ideology item is not strictly comparable to election or political polling data, it seems reasonable to infer that conservatives were underrepresented in our sample. Consequently, it is plausible that the under-representation of conservative participants in our sample may have resulted in an underestimate of the mean level of belief in conspiracy theories in the general Australasian population. While our study was intended to investigate changes in belief over time rather than prevalence of beliefs in the general population, this still presented an indirect threat to the validity of our findings about within-person variation. As reported in the main text, participants with higher (mean) levels of belief in conspiracy theories tended to demonstrate more within-person variability. As such, the under-representation of conservatives in our sample and resulting low estimated mean level of belief in conspiracy theories might have in turn resulted in our estimates of the quantity of within-person/within-person change being biased downward.

To address this possibility, we repeated several of our main analyses within each of the political ideology subsamples.

## Within-Person Standard Deviations by Political Ideology

In the main text, we report an analysis where we calculated a conspiracy score for each person at each time point, and then calculated the standard deviation of these scores over time for each participant. This analysis suggested that within-person standard deviations in scores were higher for moderates and conservatives than liberals (see Supplemental Table 7). In other words, conservatives and moderates displayed somewhat more change over time than liberals. This said, the average within-person standard deviation was small in all three groups (0.20 or less for a scale with a possible range of 1 to 5).

Supplemental Table 7
*Within-person Standard Deviations by Political Ideology*

| Ideological group | *M* of *SD_within_* |
| --- | --- |
| Liberal | 0.13 |
| Moderate | 0.19 |
| Conservative | 0.20 |
| Other | 0.20 |

*Notes*. A belief in conspiracy theories score was calculated for each participant by taking the mean of their responses to all items over all time points. Possible score range: 1 – 5.

## Intraclass Correlation Coefficient (ICC) by Political Ideology

The analyses presented above provide some evidence that there was more intra-individual/within-person variance for conservatives and moderates than liberals. However, they suggest that there was more between-person variance in these groups also. We therefore calculated the ICC for conspiracy scores (being the ratio of between-person to total variance) within the four political ideology groups separately. The results are presented in Supplemental Table 8.

This analysis was crucial, because the ICC is a key statistical result from our manuscript, and the basis for the illustration of impacts on statistical power analysis presented above. In this analysis, the ICC estimates transpired to be very similar across the four groups. Furthermore, the 95% confidence interval for each group was very narrow (i.e., < 0.1), indicating that these estimates were relatively precise. This precision is reflective of the number of data points per person, and the fact that (being a proportion) an ICC closer to 1 has relatively little sampling error.

Supplemental Table 8
*Intraclass Correlation Coefficient (ICC) by Political Ideology*

|  |  | 95% CI | |
| --- | --- | --- | --- |
| Ideological group | *r_icc_* | Lower | Upper |
| Liberal | .91 | .89 | .92 |
| Moderate | .88 | .85 | .90 |
| Conservative | .93 | .90 | .96 |
| Other | .93 | .87 | .97 |

## Implications for Credibility of Study

The analyses pertaining to political ideology suggest that some of the absolute measures of within-person change in our main manuscript may serve as mildly biased estimates of their respective population parameters due to the underrepresentation of conservatives in our sample. However, the ICC did not differ substantially across ideology groups, suggesting that our estimates of the ICC for the overall sample would be unlikely to change substantially had a sample more balanced on political ideology been recruited.

# Beliefs in Warranted Conspiracy Theories

Most of the items we included in our survey concern *unwarranted* conspiracy theories—theories which are not supported by empirical evidence (to our knowledge). However, we deliberately included two conspiracy theories that are warranted by empirical evidence. These items were included partly out of substantive interest in the degree to which belief in “warranted” conspiracy theories vary over time, and partly to maintain participants’ attention via providing some diversity of content. To identify well-known warranted conspiracy theories that met the remaining item criteria described above we relaxed the rule that theories had to concern claimed events either ongoing or in the last 20 years. The two items were:

- *Watergate*: “The administration of US President Richard Nixon covered up its involvement in the 1972 break-in of the Democratic National Committee headquarters at the Washington DC Watergate office building” ^see 15,16^.
- MKUltra: “In the 1950s, the CIA conducted illegal experiments to develop mind-altering drugs to be used to force confessions” ^see 17,18^.

Because these items represent a qualitative different form of content than the other (unwarranted) conspiracy theories, we did not include them in the analyses reported in the main manuscript. Versions of most of our major analyses pertaining to these items are instead reported below.

## Between-person Variation

We calculated each participant’s mean response across both items, and all waves. These participant means ranged from 1.00 to 5.00, with a grand mean of 3.38 (i.e., a slight tendency toward agreement). The standard deviation of the mean response across participants was 0.93, suggesting a very high degree of between-person variation.

## Change in Mean Agreement Over Time

In our next analysis, we examined changes in beliefs over time at the level of the overall sample. The mean level of agreement with each conspiracy theory at each time point is displayed in Supplementary Figure 4. As is clear in the graph, there was relatively little change in average levels of conspiracy beliefs over time. That said, there was a crossover in agreement over time: Participants initially agreed more with the MKUltra theory, but switched to agreeing (very slightly) more with the Watergate theory from time 3 onwards.

Supplementary Figure 4
*Mean Belief in Warranted Conspiracy Theories over Time*


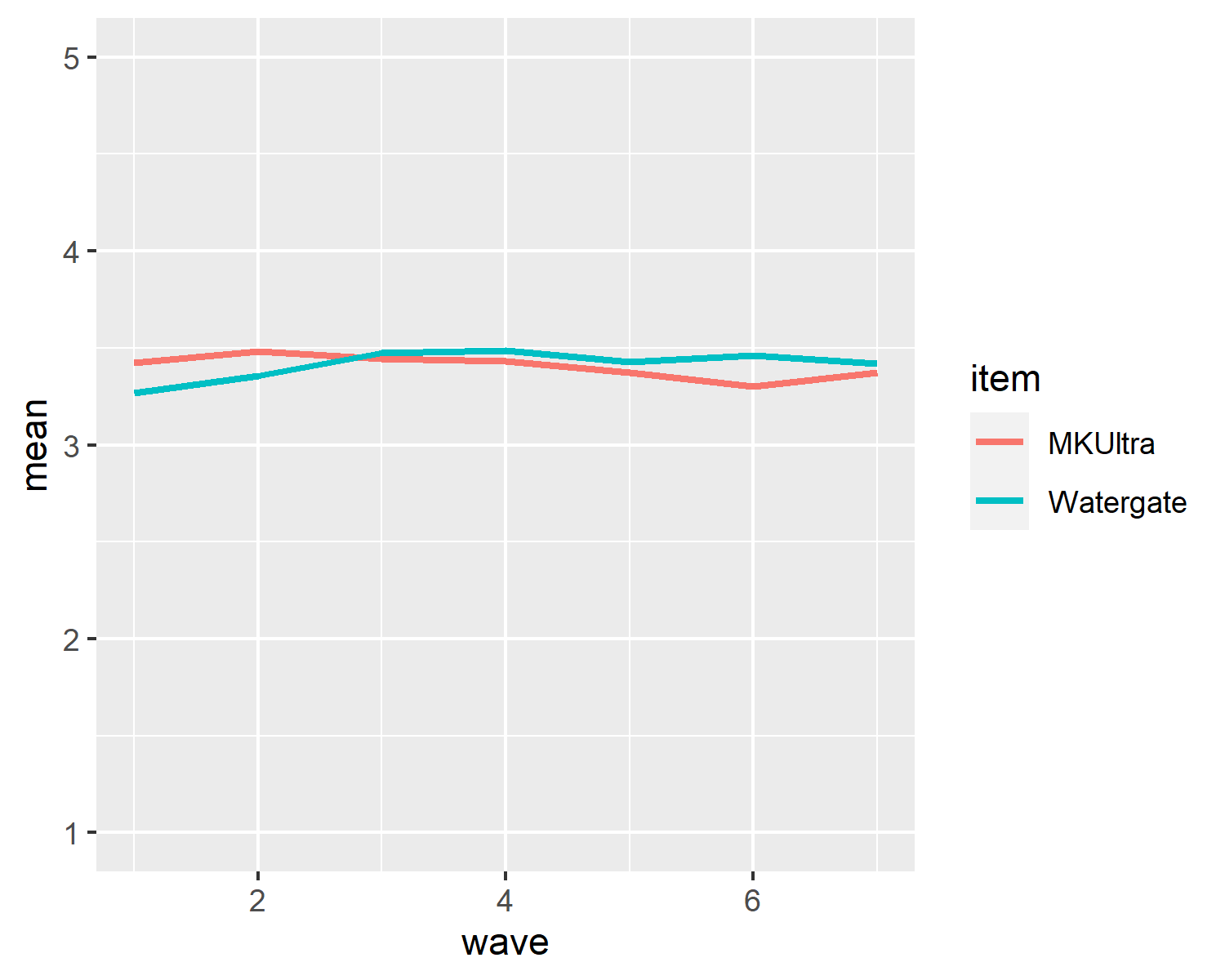


## Ranges of Belief Within Participants and Theories

For every participant and conspiracy theory we calculated the range (maximum - minimum) of responses to the two warranted conspiracy theories over time. This provided a simple indication of the degree of within-person variation. Only the 472 participants who responded to at least two waves were included in this analysis.

35.5% of the 944 ranges thus calculated were zero, indicating no change in agreement over the study. However, for 29.9% of the combinations of participant and conspiracy theory, the participant’s highest level of agreement was at least two points greater than their lowest. Two points is a relatively substantial difference in this context, indicating a change from strong [dis]agreement (1) to neither agree nor disagree (3), or from agreement (4) to disagreement (2), or vice versa. We thus used a difference of two points as a threshold for a practically significant change. A bar plot demonstrating the frequency of ranges is presented in Supplementary Figure 5.

Supplementary Figure 5

*Proportion of Belief Changes Across Beliefs*


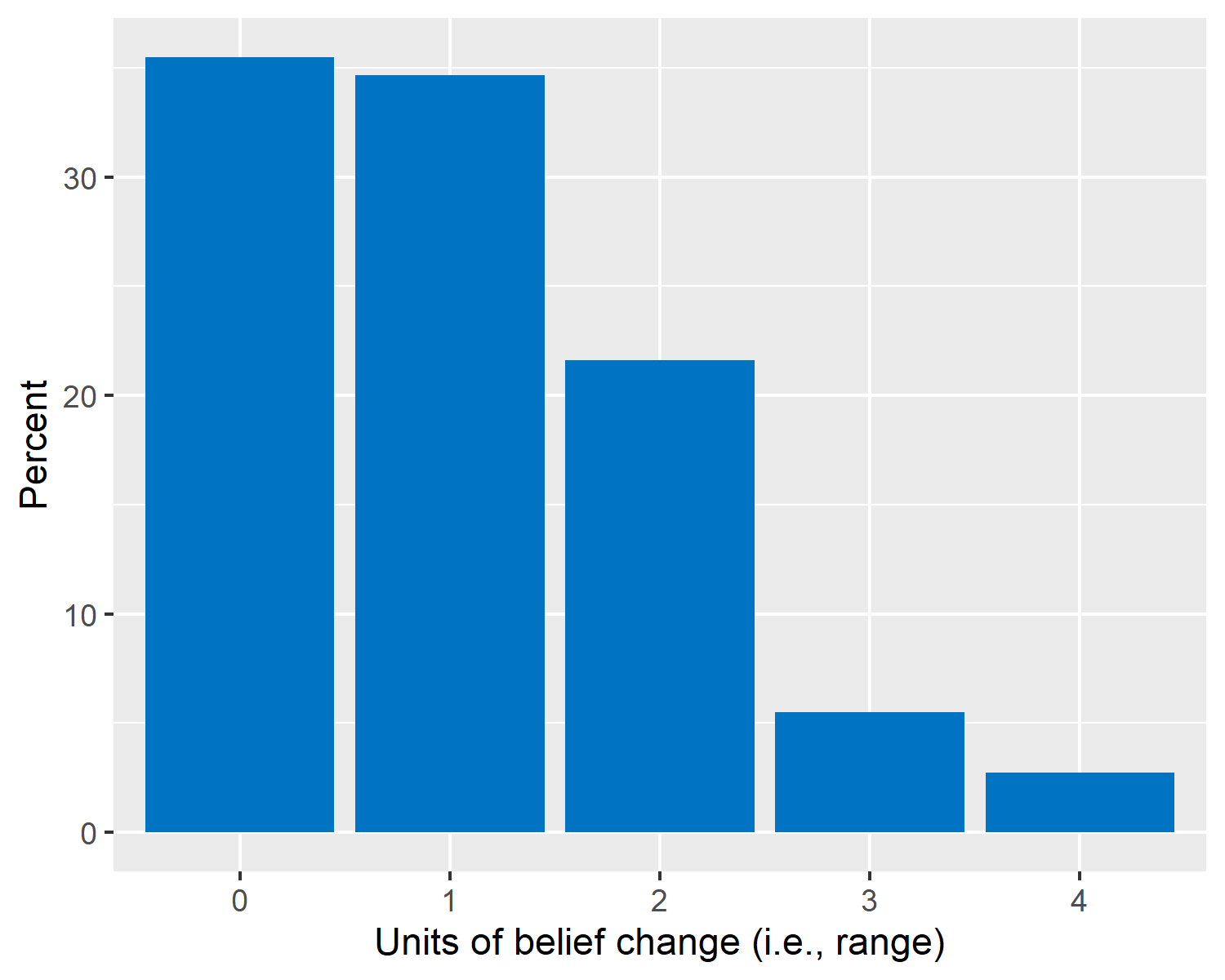


## Trajectories of Change and Stability

Trajectories were calculated using the same methods and definitions as described for the unfounded conspiracy theories in the main manuscript (see Supplementary Table 9). Notably, many participants remained consistently sceptical of these theories throughout the study.

Supplementary Table 9
*Percentage of Participants for each Trajectory*

| Trajectory | Percent (MKUltra) | Percent (Watergate) |
| --- | --- | --- |
| Consistent sceptic | 26.0 | 41.7 |
| Bump | 9.7 | 8.8 |
| Convert | 14.2 | 13.3 |
| Apostate | 13.9 | 3.3 |
| Dip | 10.0 | 4.2 |
| Consistent believer | 26.3 | 28.7 |

## Intraclass Correlation Coefficient (ICC)

A warranted conspiracy theory score was calculated for each person by taking the mean of their responses to the two items at each time point. The intraclass correlation coefficient for this score was 0.77, 95% CI [.75, .80]. This suggested that the proportion of variation in scores over time explained by individual differences was slightly lower than was the case for the unfounded conspiracy theory scores (i.e., as reported in the main manuscript.

## Accounting for Measurement Error

We do not report a structural equation model for the true theories in isolation since the inclusion of just two items per within-person factor makes the model unidentifiable.

## Correlation between Beliefs in Warranted and Unfounded Conspiracy Theories

As a supplementary analysis, we examined the relationship between beliefs in warranted and unfounded conspiracy theories. To do this, we obtained an unfounded conspiracy theories score for each participant by taking the mean of all their responses to the ten unfounded conspiracy theory items over time. We then created a warranted conspiracy theories score by taking the mean of all their responses to the two warranted conspiracy theory items over time. The correlation between these two scores was *r* = .12, 95% CI [.03, .20]. This suggests that participants who believe in unfounded conspiracy theories are more likely to agree with warranted conspiracy theories, but this relationship is small.

# Predictors of Completing More Waves

As mentioned in the main text, there was substantial attrition in this study: Although 95% of participants completed at least two waves, just under half (49.8%) completed all seven (see Supplementary Table 10). It is therefore potentially useful to consider which variables (collected at time 1) predicted completing more waves. This analysis could be useful to future researchers seeking to recruit participants for longitudinal studies using platforms such as Prolific. It also provides some information about the characteristics of participants who contributed most data to the study, and thus most strongly drive the findings.

In terms of selecting an appropriate statistical model, it is relevant to consider that the study had seven waves (which we could conceptualise as “trials”). Every participant could choose to participate in a given wave, or not (i.e., a binary outcome at each trial). An appropriate statistical model is therefore a binomial logistic regression model with multiple trials. This is a generalisation of the familiar special case of the binomial logistic regression where the outcome variable is dichotomous (one trial).

Supplementary Table 10
*Summary of Attrition*

| Number of waves responded to | *n* of participants | Percent of original sample (*N* = 498) |
| --- | --- | --- |
| 7 | 248 | 49.8 |
| 6 or more | 323 | 64.9 |
| 5 or more | 371 | 74.5 |
| 4 or more | 401 | 80.5 |
| 3 or more | 434 | 87.1 |
| 2 or more | 472 | 94.8 |
| 1 or more | 498 | 100.0 |

## Time 1 Survey Data

In the first model, we predicted waves completed using variables from our time 1 survey. The variables we used as predictors were the demographic variables we collected (age, country, gender, education level, political ideology/orientation, employment status, marital status) along with a total score on the conspiracy theory scale. As mentioned above, we fit a binomial logistic regression model with multiple trials.

The parameter estimates for this model are displayed in Supplementary Table 11. One notable finding in the table is that age was a strong positive predictor of completing more waves. The model estimates that there is a 5.7% increase in the odds of completing any given wave for each extra year of age, holding the other predictors constant. This is important considering that Prolific participants tend to be quite young, and may imply that deliberately recruiting samples that are more representative of the general population in terms of age may be useful for avoiding attrition. Another significant predictor was conspiracy theory score: The model estimates that there is a 34.6% increase in the odds of completing any given wave for each one-unit increase on the conspiracy theory scale (which has a possible range 1 to 5), when holding the other predictors constant. In other words, participants with higher levels of belief in conspiracy theories tended to be *more* likely to return for subsequent waves. This is a reassuring finding given the importance of participants with higher levels of belief in conspiracy theories in this study.

Supplementary Table 11
*Binomial Logistic Regression Model Predicting Waves Completed from Time 1 Survey Data*

|  |  |  |  | 95% CI for Exp(*b*) | |
| --- | --- | --- | --- | --- | --- |
|  | Estimate (*b*) | *p* | Exp(*b*) | Lower | Upper |
| (Intercept) | -0.525 | .156 | 0.591 | 0.288 | 1.232 |
| Age (in years) | 0.055 | < .001^*^ | 1.057 | 1.043 | 1.071 |
| Country^1^ (New Zealand) | 0.294 | .012^*^ | 1.341 | 1.070 | 1.693 |
| Gender^2^ (female) | -0.018 | .850 | 0.982 | 0.817 | 1.181 |
| Gender (gender diverse / non-binary) | 0.087 | .847 | 1.091 | 0.470 | 2.843 |
| Education^3^ (postgraduate) | -0.514 | .046^*^ | 0.598 | 0.354 | 0.973 |
| Education (undergraduate) | -0.320 | .193 | 0.726 | 0.438 | 1.153 |
| Education (other tertiary) | -0.802 | .002^*^ | 0.448 | 0.263 | 0.735 |
| Education (completed high school) | -0.657 | .014^*^ | 0.519 | 0.301 | 0.861 |
| Education (no high school) | -0.181 | .758 | 0.834 | 0.287 | 3.052 |
| Political^4^ (moderate) | -0.209 | .036^*^ | 0.811 | 0.668 | 0.986 |
| Political (conservative) | -0.260 | .158 | 0.771 | 0.540 | 1.113 |
| Political (other) | 0.492 | .079 | 1.635 | 0.968 | 2.916 |
| Employment^5^ (part-time) | -0.183 | .120 | 0.833 | 0.663 | 1.049 |
| Employment (unemployed) | -0.477 | .003^*^ | 0.621 | 0.455 | 0.850 |
| Employment (homemaker) | 0.161 | .524 | 1.175 | 0.728 | 1.967 |
| Employment (student) | -0.030 | .839 | 0.970 | 0.725 | 1.302 |
| Employment (retired) | -0.140 | .825 | 0.870 | 0.290 | 3.762 |
| Employment (other) | -0.384 | .105 | 0.681 | 0.432 | 1.098 |
| Marital^6^ (living with partner) | -0.090 | .509 | 0.914 | 0.700 | 1.195 |
| Marital (widowed) | -1.645 | .112 | 0.193 | 0.026 | 1.763 |
| Marital (divorced/separated) | -0.035 | .900 | 0.966 | 0.573 | 1.699 |
| Marital (never married) | 0.477 | < .001^*^ | 1.612 | 1.240 | 2.096 |
| Conspiracy theory score (T1)^7^ | 0.297 | < .001^*^ | 1.346 | 1.162 | 1.564 |

*Notes.* ^*^*p* < .05.
^1^Current country of residence. Reference level: Australia.
^2^Reference level: Male.
^3^Highest level of completed education. Reference level: Doctoral.
^4^Political orientation/ideology. Reference level: liberal.
^5^Employment status. Reference level: In full-time work.
^6^Marital status. Reference level: Married.
^7^Mean of participants’ responses to the ten unfounded conspiracy theory items at time 1. Possible range: 1 to 5.

## Prolific Participation Variables

In the second model for predicting retention, we considered variables derived from the participant demographic data provided by Prolific when a study is run using the platform. The predictive value of these variables is pertinent because future researchers could use them in prescreening criteria on Prolific. However, unlike all our other analyses, these analyses are not reproducible in our open data; while this data from Prolific is not identifiable, we do not have explicit permission from participants to share it.

The demographic data provided by Prolific has a number of variables in it, most of which did not appear to be suitable for inclusion in our predictive model. These variables include:

- Variables describing the participant’s completion of our time 1 survey, including session ID, completion status, started time, completed time, and time taken/duration. These variables do not describe the participants themselves and could not be used as pre-screeners.
- Demographic variables with very large quantities of missing data (“data expired” in Prolific’s terminology); these include student status and employment status.
- Age and sex. Barring the distinction between gender (the term used in our survey) and sex, these are essentially duplicated demographic variables whose predictive value we have already assessed above.
- Country of birth, nationality, and current country of residence. Given our restriction of participants to current residents of Australia and New Zealand, these variables were all dominated by Australians and New Zealanders, albeit with small numbers of participants having a nationality or country of birth from each of a wide range of other countries. Including these variables as factor predictors would thus make for an overparameterised model that would be unlikely to generalise well. (Differences in attrition between Australians and New Zealanders have already been investigated above; see
- Supplementary Table 11).
- First language. This variable as likewise dominated by speakers of English as a first language, with a small number of first-language speakers of each of a wide range of other languages. Again, including this variable would make for an overparameterised model vulnerable to overfitting.

With these variables ruled out as potential predictors, this left variables reflecting participants’ prior participation on Prolific: the number of approved submissions, number of rejected submissions, and “Prolific score”. Prolific indicates that the Prolific score reflects “a participant’s approval rate across all studies they have taken part […] It is essentially a percentage score - so if a participant is at 100 then 100% of their previous submissions have been approved” (personal communication, 16 July 2021). Interestingly, this definition varies from that given in Prolific’s pre-screening database, which states that “We use the upper bound of the 95% confidence interval to calculate approval rate.” In our data, the “Prolific score” does not seem to exactly equal either the percentage of approved submissions or the upper limit of its 95% confidence interval (calculated using either the normal approximation or the Clopper and Pearson method). We surmise either that Prolific uses some other inputs in the calculation (e.g., submissions not yet approved or rejected), or that it does calculate a confidence interval but via a different method than we have attempted.

In any event, to fit a predictive model we needed to deal with the fact that Prolific score is some function of the number of approvals and rejections, implying a multicollinearity problem. We therefore calculated a new variable, number of submissions as the sum of approvals and rejections for each participant. This variable also has the advantage of corresponding to the “number of previous submissions” prescreening criterion in Prolific (number of rejections and number of approvals are provided in the demographic data but not available as pre-screeners per se). We then entered this variable into a binomial logistic regression model along with Prolific score.

The coefficients for this model are displayed in Supplementary Table 12. The coefficient for number of prior submissions was statistically significant, but Prolific score was not. This is presumably a reflection of the very limited variance in Prolific scores (*M* = 99.47, *SD* = 1.16), despite the fact that we did not specify a Prolific score cut-off as an inclusion criterion in our own study.

The log odds of 1.006 for number of prior submissions suggests that for every extra prior submission, the probability of a participant responding to a given wave increases by 0.6%. Superficially this may seem like a small effect, but it is quite large considering that many of our participants had participated in hundreds of studies (*M* = 254, *SD* = 204, Q_1_ = 104, Q_3_ = 349). In future longitudinal studies conducted using Prolific it may therefore be wise to specify a prescreening criterion relating to number of prior submissions, although our findings do not suggest any specific cut-off.

Supplementary Table 12
*Binomial Logistic Regression Model Predicting Waves Completed from Prior Submissions and Prolific Score*

|  |  |  |  | 95% CI for Exp(*b*) | |
| --- | --- | --- | --- | --- | --- |
|  | Estimate *b* | Exp(*b*) | *p* | Lower | Upper |
| (Intercept) | 3.457 | 31.723 | 0.321 | 0.038 | 33378.674 |
| num_submissions | 0.006 | 1.006 | <.001 | 1.006 | 1.007 |
| prolific_score | -0.034 | 0.966 | 0.330 | 0.901 | 1.034 |

*Notes.* ^*^*p* < .05

## Statistical Assumptions of Analyses Presented in Main Text

Many of the statistics reported in the results in the main text are descriptive, and should be read as applying to the sample at hand rather than as parameter estimates or tests pertaining to a larger population. It is not necessary to invoke formal assumptions for these statistics. However, in a handful of cases we do report formal statistical inferences (e.g., confidence intervals and/or *p* values). These analyses do rely on statistical assumptions.

Our analysis of the Spearman’s correlation between average levels of belief in conspiracy theories and within-person standard deviations in belief relies primarily on the assumption that the relationship between these two variables is monotonic (i.e., always positive, or always negative, if not necessarily linear). A scatter plot indicated this assumption was reasonable (see Supplementary Figure 6).

Our analyses using intraclass correlation coefficients implicitly rely on ANOVA models attributing variance to different components. They thus rely on the standard assumptions of linear/regression models estimated via ordinary least squares: that the predictors are measured without error, and that the error terms are independently, identically, and normally distributed with mean zero^19^. Given that the “predictors” in the ICC models are essentially just participant IDs, these can reasonably be assumed to be measured without error. The fact that the outcome variable in our analysis (i.e., scores on a measure of belief in conspiracy theory) was created by taking the mean responses to items with discrete response options implies that the assumption of normality of errors cannot strictly be met (since the normal distribution is continuous and unbounded). However, this is the least important assumption of a linear model; linear models are highly robust to breaches of this assumption unless the sample is very small^20,21^.

Our mixed model to test whether changes in mean belief over time in the sample as a whole were statistically significant relies on the same assumptions discussed in the paragraph above, plus the (untestable) assumption that the random effects were each normally distributed. This analysis used repeated observations from the same people, and it is possible that our specification of a random intercept across person was not quite sufficient to fully address all sources of error dependence. A model where the effect of time was permitted to vary randomly across participants and items would have been more conservative as a strategy for avoiding error dependence, but the model would not converge unless the effect of time was fixed. This issue was relatively inconsequential for our statistical inferences since error dependence would not bias the point estimate of the effect of time, but at worst result in an artificial narrowing of its confidence interval—and the confidence interval spanned zero regardless.

Our use of a structural equation model to distinguish within-person variance from measurement relies on the assumption that the model is correctly specified (e.g., that there are no other sources of covariance between variables than those specified in the model). The fact that the model fit imperfectly (as reported in the main text) suggests this assumption may have been breached. Readers should therefore treat the parameter estimates it produces as having additional uncertainty attached beyond that conveyed in the confidence intervals.

Supplementary Figure 6
*Mean Levels of Belief in Conspiracy Theories and Within-Person Standard Deviations*


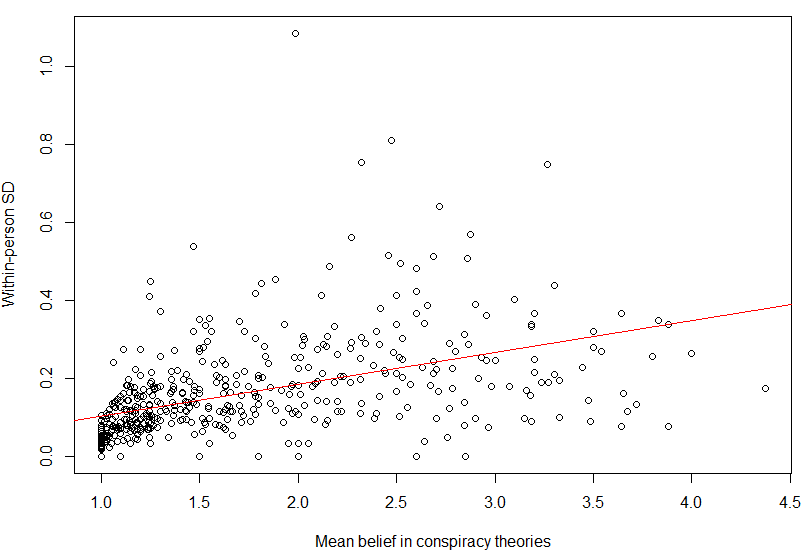


# References

1. Zou, G. Y. Sample size formulas for estimating intraclass correlation coefficients with precision and assurance. *Stat. Med.* **31**, 3972–3981 (2012).

2. Swami, V. *et al.* Conspiracist ideation in Britain and Austria: Evidence of a monological belief system and associations between individual psychological differences and real-world and fictitious conspiracy theories. *Br. J. Psychol.* **102**, 443–463 (2011).

3. Coady, D. Psychology and conspiracy theories. in *The Routledge handbook of applied epistemology* 166–176 (Routledge, 2018).

4. van Prooijen, J.-W. & Acker, M. The influence of control on belief in conspiracy theories: conceptual and applied extensions. *Appl. Cogn. Psychol.* **29**, 753–761 (2015).

5. Swami, V., Chamorro‐Premuzic, T. & Furnham, A. Unanswered questions: A preliminary investigation of personality and individual difference predictors of 9/11 conspiracist beliefs. *Appl. Cogn. Psychol.* **24**, 749–761 (2010).

6. Swami, V., Chamorro-Premuzic, T. & Furnham, A. Unanswered questions: A preliminary investigation of personality and individual difference predictors of 9/11 conspiracist beliefs. *Appl. Cogn. Psychol.* **24**, 749–761 (2010).

7. Mulder, J. D. & Hamaker, E. L. Three extensions of the random intercept cross-lagged panel model. *Struct. Equ. Model. Multidiscip. J.* **28**, 638–648 (2021).

8. Li, C.-H. Confirmatory factor analysis with ordinal data: Comparing robust maximum likelihood and diagonally weighted least squares. *Behav. Res. Methods* **48**, 936–949 (2016).

9. Hu, L. & Bentler, P. M. Cutoff criteria for fit indexes in covariance structure analysis: Conventional criteria versus new alternatives. *Struct. Equ. Model.* **6**, 1–55 (1999).

10. Hamaker, E. L., Kuiper, R. M. & Grasman, R. P. P. P. A critique of the cross-lagged panel model. *Psychol. Methods* **20**, 102–116 (2015).

11. Bierwiaczonek, K., Kunst, J. R. & Pich, O. Belief in COVID-19 conspiracy theories reduces social distancing over time. *Appl. Psychol. Health Well-Being* **12**, 1270–1285 (2020).

12. Liekefett, L., Christ, O. & Becker, J. C. Can conspiracy beliefs be beneficial? Longitudinal linkages between conspiracy beliefs, anxiety, uncertainty aversion, and existential threat. *Pers. Soc. Psychol. Bull.* **49**, 167–179 (2023).

13. Mulder, J. powRICLPM: Power analysis for the random intercept cross-lagged panel model. (2021).

14. Sutton, R. M. & Douglas, K. M. Conspiracy theories and the conspiracy mindset: Implications for political ideology. *Curr. Opin. Behav. Sci.* **34**, 118–122 (2020).

15. Bernstein, C. & Woodward, B. *All the President’s men*. (Simon & Schuster, 1974).

16. Watergate scandal. *Wikipedia* (2022).

17. Committee on Human Resources. *Project MKUltra, the CIA’s program of research in behavioral modification*. https://info.publicintelligence.net/SSCI-MKULTRA-1977.pdf (1977).

18. Project MKUltra. *Wikipedia* (2022).

19. Williams, M. N., Grajales, C. A. G. & Kurkiewicz, D. Assumptions of multiple regression: Correcting two misconceptions. *Pract. Assess. Res. Eval.* **18**, (2013).

20. Gelman, A. & Hill, J. *Data analysis using regression and multilevel/hierarchical models*. (Cambridge University Press, 2007).

21. Lumley, T., Diehr, P., Emerson, S. & Chen, L. The importance of the normality assumption in large public health data sets. *Annu. Rev. Public Health* **23**, 151–169 (2002).
